# Toward Point-of-Care Drug Quality Assurance in Developing Countries: Comparison of Liquid Chromatography and Infrared Spectroscopy Quantitation of a Small-Scale Random Sample of Amoxicillin

**DOI:** 10.4269/ajtmh.17-0779

**Published:** 2018-06-11

**Authors:** Norah Alotaibi, Sean Overton, Sharon Curtis, Jason W. Nickerson, Amir Attaran, Sheldon Gilmer, Paul M. Mayer

**Affiliations:** 1 Department of Chemistry and Biomolecular Sciences, University of Ottawa, Ottawa, Ontario, Canada;; 2 Bruyère Research Institute, Ottawa, Ontario, Canada;; 3 Faculty of Law, University of Ottawa, Ottawa, Ontario, Canada;; 4 World Hope Canada, Kemptville, Ontario, Canada

## Abstract

Substandard antibiotics are thought to be a major threat to public health in developing countries and a cause of antimicrobial resistance. However, assessing quality outside of a laboratory setting, using simple equipment, is challenging. The aim of this study was to validate the use of a portable Fourier transform infrared (FT-IR) spectrometer for the identification of substandard antibiotics. Results are presented for amoxicillin packages from Haiti, Ghana, Sierra Leone, Democratic Republic of Congo, India, Papua New Guinea, and Ethiopia collected over the course of 6 months in 2017, including two field trips with the FT-IR to Ghana and Sierra Leone. Canadian samples were used as a control. Regarding drug quality, of 290 individual capsules of amoxicillin analyzed, 13 were found to be substandard with total active pharmaceutical ingredients (API) lying outside the acceptable range of 90–110%. Of these 13, four were below 80% API. The FT-IR reliably identified these outliers and was found to yield results in good agreement with the established pharmacopeia liquid chromatography protocol. We conclude that the portable FT-IR may be suitable to intercept substandard antibiotics in developing countries where more sophisticated techniques are not readily available.

## INTRODUCTION

In recent years, evidence has emerged that the problem of substandard medicines is large and growing.^1^ These medicines, which may contain the wrong amount or type of ingredients, can cause drug resistance, injury, and death—sometimes hundreds of patients at a time. Little is known about the true dimensions of this issue and which patients or medicines it targets, although work by the World Health Organization (WHO) and INTERPOL roughly estimates that it affects between 1% and 30% of drugs.^2^ Loopholes in law and regulation let wrongdoers trade bad-quality drugs, with little risk of punishment. INTERPOL notes that police reports of medicine crime are up 10-fold since 2000, sometimes causing hundreds of deaths.^3^ Serious as that is, WHO laments that current data on medicine quality “are often of poor quality and are not … systematized.”^2^ Pezzola and Sweet, in a study of developing indicators for pharmaceutical regulation in developing countries, have noted resistance to the implementation of quality standards.^4^ Even neighboring countries can have wildly different levels of regulation.^4^ Our team has corroborated these concerns in small-scale exploratory studies that clearly illustrate the grave risks of treatment failure, drug resistance, and death. For example, we linked a specific vial of substandard antibiotic ceftriaxone in a Ugandan hospital to a young boy’s death in that hospital.^5^ In addition, we recently published on the identification of substandard propofol in Zambia, which was identified because of concerns of drug quality following several adverse events on administration, ranging from bronchospasm to hypotension and cardiac arrest.^6^ The literature contains little data on medicine testing in most disease areas (e.g., almost no published studies of noncommunicable disease drugs), which in turn means there is not an evidence base that politicians find compelling enough to strengthen the laws and law enforcement needed to effectively criminalize and punish those who traffic substandard medicines. Nayyar and coworkers^7,8^ published reviews in 2012 and 2015, highlighting the significance of falsified pharmaceuticals worldwide and the detection strategies that have been used. In addition to the high-performance liquid chromatography (HPLC) pharmacopeia standard methods, colorimetric and thin-layer chromatographies are two of the more common, less expensive, testing methods used.

A limitation of most studies in the literature is that they are carried out ex post facto, that is, after a patient or patients have been harmed by substandard dosing (as was performed in our previous work). This is a consequence of gold standard quantitative analysis being laboratory-based, and it points to a need for more portable, cost-effective, and user-friendly methods if this type of analysis is to be performed in a preventative manner.^9^ Portable near-infrared (IR) and Raman spectroscopy devices have been deployed with mixed success, with quantitation of the active pharmaceutical ingredient (API) being most challenging.^9–14^ For example, IR spectroscopy has been used to probe paracetamol tablets, over the fingerprint region 1,100–2,400 cm^−1^.^15^ The authors determined that once a database of drugs had been developed, it was possible to demonstrate the reliability of a sample quickly and efficiently.

We have chosen to use Fourier transform IR (FT-IR) spectroscopy over an extended wavelength range to better capture the IR spectrum and identify changes to excipient composition, thus hopefully limiting false negatives and positives. To this end, we have partnered with Agilent Technologies, Inc (Lexington, MA). to develop one of their portable FT-IR spectrometers for the identification of substandard antibiotics. In this report, we focus on the results obtained with the FT-IR and standard laboratory testing for amoxicillin capsules collected over the course of 6 months from Haiti, Ghana, Sierra Leone, Papua New Guinea, the Democratic Republic of Congo (DRC), India, and Ethiopia. The pharmacopeia standard HPLC method was also used to serve as a validation for the FT-IR results, especially because the IR spectra are sensitive to excipients found in the capsules.

## MATERIALS AND METHODS

### Sampling.

A total of 57 blister packages of amoxicillin, representing 37 batch numbers containing 250 and 500 mg capsules were purchased from commercial outlets in eight countries. Twelve packages were obtained in the DRC (Kinshasa), 11 in Ghana (Accra and Tema), 21 in Sierra Leone (Freetown, Makeni and Kamakwie), seven in Haiti (Port-au-Prince), three in Ethiopia (Addis Ababa), and one each in Papua New Guinea (Port Moresby), India (New Delhi), and Canada (Ottawa, to serve as a control). Packages from Ghana and Sierra Leone were collected during a 2-week trip to those countries with the portable FT-IR. All samples were unexpired. A complete list of all packages and their origin can be found in Supplemental Table 1. Samples from the DRC were obtained from the central government depot, whereas the rest were purchased from private pharmacies.

### Sample preparation.

Five capsules were removed from each blister pack (10 from the package from Canada) and analyzed separately (and reported as discrete data points for a total of 290 capsules measured). Capsule content weight was determined by calculating the difference between the total weight of the capsule and the empty capsule. For each capsule, two 30-mg aliquots of contents were dissolved in 50 mL of the mobile phase (see the following paragraphs) and filtered before analysis via ultra-high-performance liquid chromatography (UPLC)–ultraviolet (UV)/visible spectroscopy (UPLC-UV for short). The remainder of the capsule content was used for FT-IR analysis. Each capsule was subjected to duplicate measurements by UPLC-UV and triplicate measurements by FT-IR, with the averages being reported in the figures and table.

### Ultra-high-performance liquid chromatography-ultraviolet protocol.

The UPLC-UV analysis was performed in accordance with the established pharmacopeia protocol^16^ on an Agilent 1290 Infinity II system equipped with the following: the G7120A high-speed binary pump, the G7167B multisampler with a 25-μL sample loop, the G7116B multicolumn thermostat, and the G7114B variable wavelength detector (VWD). Reagents were purchased from Sigma-Aldrich and acetonitrile was purchased from Fisher Scientific Canada (Ottawa, Ontario). Amoxicillin trihydrate, pharmaceutical secondary standard, a certified reference material traceable to BP 19; PhEur A0900000; and USP 1031503 was purchased from Sigma-Aldrich. Standard calculations (Sigma-Aldrich, Oakville, Ontario, https://www.sigmaaldrich.com/analytical-chromatography/hplc/method-transfer-calculator.html) were used to translate the HPLC pharmacopeial procedure to UPLC (which only changes mobile phase flow rate and column diameter). The mobile phase used was 0.2 M American Chemical Society (ACS)-grade potassium phosphate monobasic at a pH of 5.0, adjusted with 2 M of ACS-grade sodium hydroxide, and UPLC-UV–quality acetonitrile at an isocratic ratio of 87:13. The flow rate for the mobile phase was 0.579 mL/minute. The stationary phase used was the Zorbax SB-C18 UPLC column with a column length of 0.05 m, an internal diameter of 2.1 mm, and a pore size of 1.8 μm. An injection volume of 10 μL was used and the VWD was set at 254 nm.^16^

Three aliquots of certified reference material–grade amoxicillin trihydrate (Sigma-Aldrich) was weighed in 15, 30, and 45 mg amounts and then dissolved in 50 mL of mobile phase and filtered. The standard was analyzed at the same time as the analyte sample to produce the external calibration curve for quantitation. The amount of amoxicillin in the analyzed aliquot interpolated from the calibration curve is converted to the relative amount of API in %w/w and multiplied by the total weight of the capsule’s contents.^16^ A complete set of numerical results and their uncertainty can be found in Supplemental Table 1.

### Fourier transform infrared protocol.

In triplicate, approximately 30–50 mg from each capsule was placed on the FT-IR sampling crystal of an Agilent 4500a portable FT-IR equipped with a single-bounce attenuated total reflectance sampling accessory. Both background and sample analyses are measured at a resolution of 4 cm^−1^. One hundred and twenty-eight scans were performed over the spectral range of 4,000–650 cm^−1^ for a total run time of 2 minutes.

The calibration curve of %w/w of amoxicillin (12 points varying between 0% and 84.6% of anhydrous amoxicillin) was prepared by varying the amount of amoxicillin trihydrate (see UPLC protocol for information on this standard) in a capsule and filling them to 594 mg with an excipient matrix as described in the literature.^17^ Ten grams of excipient matrix was prepared by mixing 4.997 g of sodium dodecyl sulfate, 4.330 g of magnesium stearate, and 0.673 g of silicon dioxide. All excipients were of ACS grade and purchased from Sigma-Aldrich. The standards were then run in triplicate and a partial least squares multiple linear regression calibration model was made using Agilent’s MicroLab Expert with the area selected in the region of 1,800–1,150 cm^−1^. Examples of the IR spectrum of amoxicillin and the calibration mixtures are found in Supplemental Figure 1, together with a calibration curve taken at a single wavelength to demonstrate linearity. The software gives the relative percentage of weight to weight of the API. Multiplying this percentage by the total mass of the sample yields the total mass of the API. Dividing the total mass of API by the expected mass from the package label results in the percentage API (%API) used in the figures. A complete set of numerical results and their uncertainty can be found in Supplemental Table 1.

## RESULTS AND DISCUSSION

### Total dose mass.

**Figure 1.**
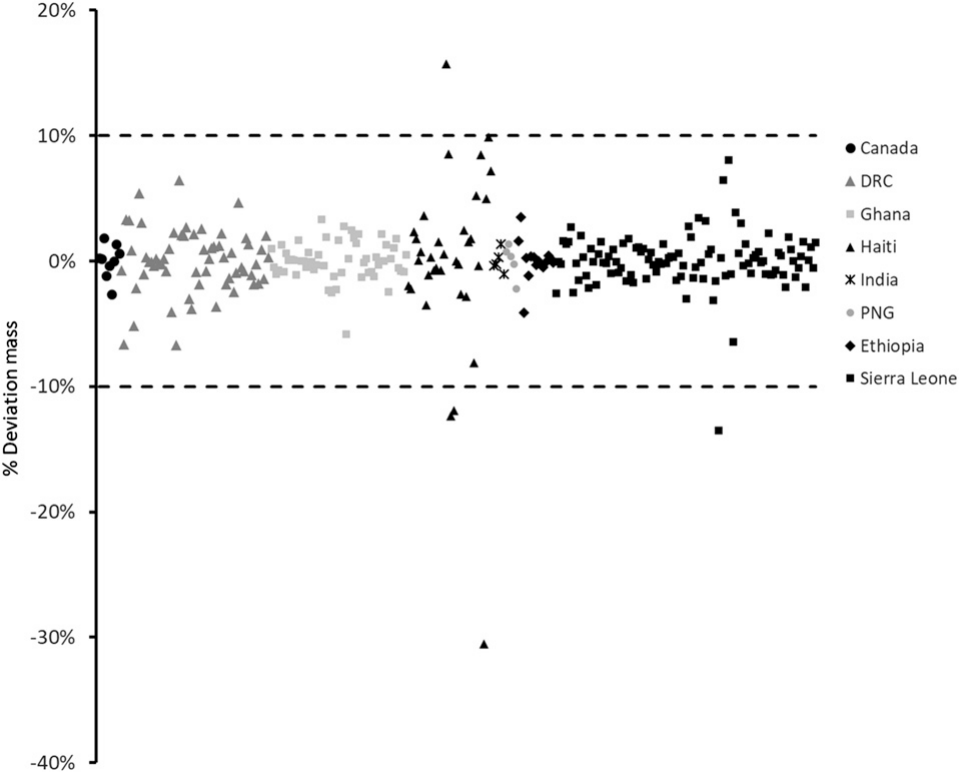
Plot of the % deviation (%D) in content mass from each capsule, as a function of country of origin. The dashed lines represent the allowable %D.

The simplest analysis performed on the capsules was to weigh them. This allowed for both the identification of outliers as well as the determination of the %API by FT-IR (see methodology). The results across all samples, plotted by country of origin, are shown in Figure 1 as the % relative standard deviation from the expected mass listed on the packaging. Individual results are listed in Excel spreadsheet format in Supplemental Text. All but five of the measurements fall within the allowable ±10% of the listed mass, with a few exceptions. Notably, three of the capsules obtained from Haiti were significantly low. This points to poor quality control in the capsule filling process and leads to low values for the %API (see the following paragraphs).

### Ultra-high-performance liquid chromatography-ultraviolet results.

**Figure 2.**
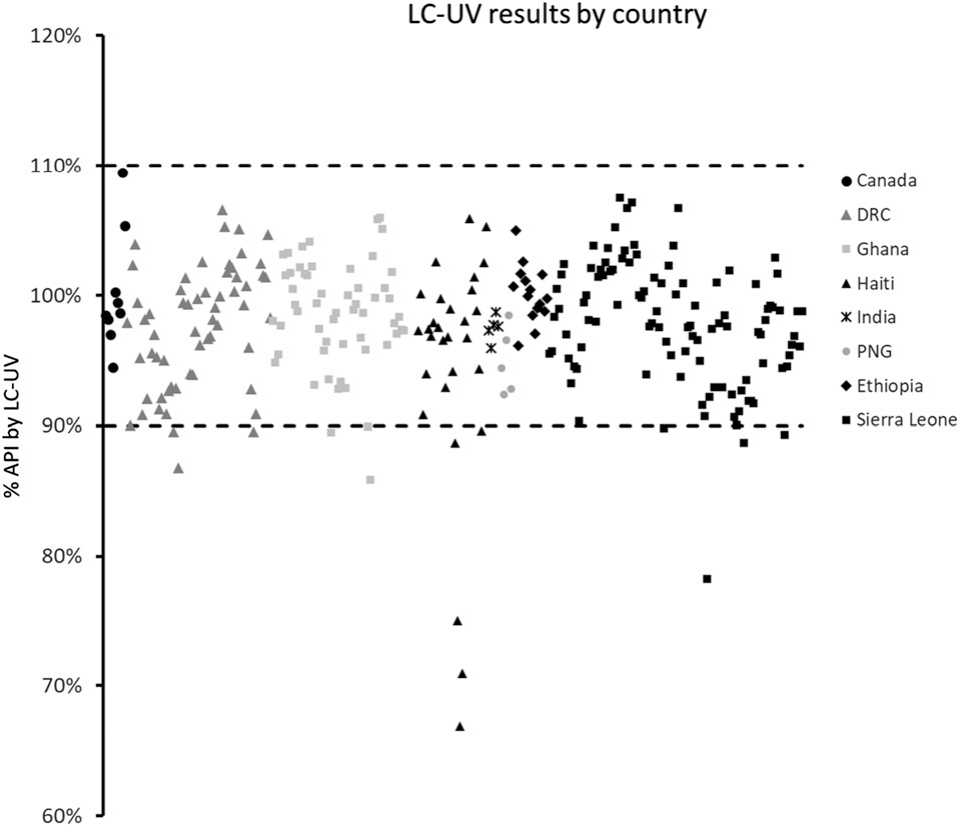
Plot of the % active pharmaceutical ingredients (API) for each capsule as determined by liquid chromatography–ultraviolet (LC–UV) analysis, as a function of country of origin. The dashed lines represent the acceptable limits in percentage API (%API).

The results of the %API across all samples are summarized in Figure 2 as a function of country of origin. Ninety-five percent of the capsules fell within the 90–110% limit for the %API, but 13 fell below that deemed acceptable. As expected, those capsules with very poor content masses by extension also had very low %API. Of particular concern were selected samples from Haiti, the DRC, Ghana, and Sierra Leone that fell well below 90% API, including one capsule from Haiti that contained only 65% API.

### Fourier transform infrared results.

**Figure 3.**
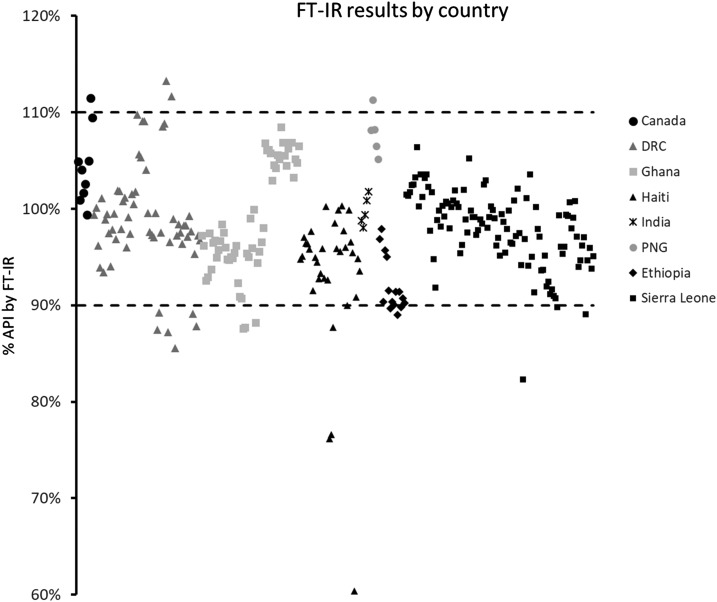
Plot of the % active pharmaceutical ingredients (API) for each capsule as determined by Fourier transform infrared (FT-IR) analysis, as a function of country of origin. The dashed lines represent the acceptable limits in percentage API (%API).

The results derived from the FT-IR protocol of the %API across all capsules are summarized in Figure 3 as a function of country of origin. As was seen in the UV results, most capsules (92%) fall inside the approved guidelines of 90–110% API. Eight percent of the capsules fell outside this range; however, all but four lie close to the 90% API cutoff.

**Figure 4.**
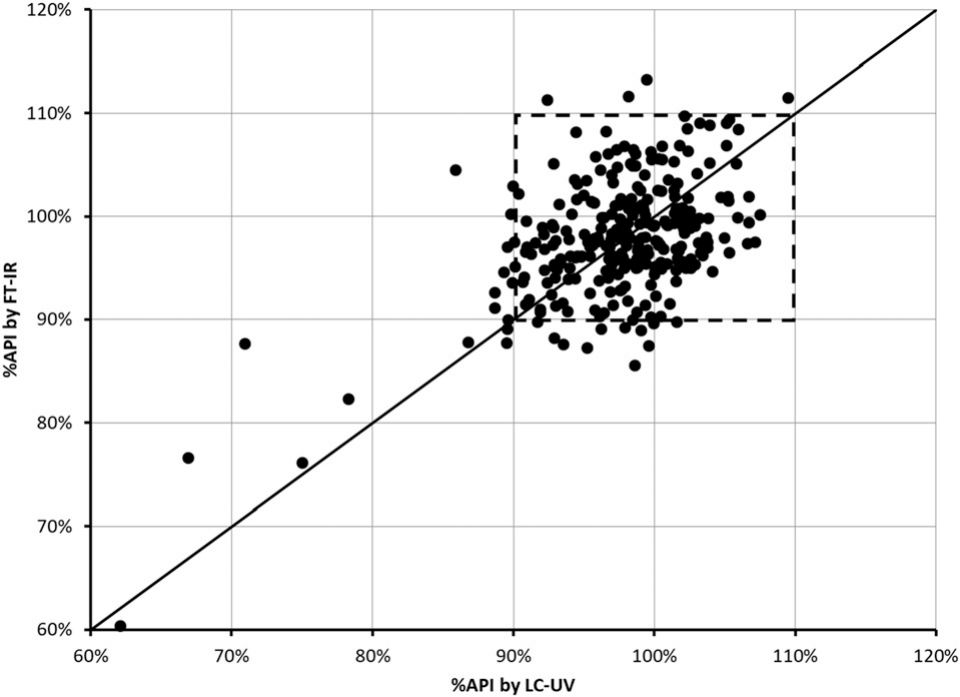
Plot of the % active pharmaceutical ingredients (API) for each capsule as determined by Fourier transform infrared (FT-IR) analysis compared with that from liquid chromatography–ultraviolet (LC-UV). The solid line represents 1:1 agreement. The dashed box highlights the area of acceptable percentage API (%API).

Figure 4 displays the %API found for each capsule by the two techniques. The solid line represents the ideal 1:1 agreement between the two. There is generally good agreement between the two methods with the data clustering along the 1:1 line. The FT-IR predicts low %API values for the same capsules identified by LC-UV. In other words, the major outliers, and thus the capsules of concern, are identified by both techniques.

### Analysis cost.

The cost to analyze a single package by UPLC-UV, comprised of five randomly selected capsules, was $34 USD, which takes into account solvents, standards, and disposables (i.e., syringes, filter tips, etc.). The total amount of time needed was 4.75 hours (primarily because of sample preparation). In comparison, the FT-IR has no consumables cost and required only 30 minutes to analyze the same five capsules once the method has been validated. Not factored into this is the difference in cost of the instruments themselves, with the FT-IR significantly lower in cost than an LC-UV system.

## CONCLUSION

Regarding drug quality, 13 of 290 individual capsules of amoxicillin were found to be substandard with total API lying outside the acceptable range of 90–110%, with five of these being below 80%. Although not the focus of this study, a simple power calculation indicates that the 4.4% substandard samples is statistically significant (the error due to sample size in this case being 2.4%). The FT-IR reliably identified these outliers and was found to yield results in good agreement with the established pharmacopeia UPLC-UV protocol. Matrix effects were clearly identifiable in the FT-IR since the entire IR spectrum is being acquired, rather than a single wavelength. This avoids false readings from entering into the results. It is possible to modify the spectral window used in the calibration and analyses after the fact, without having to rerun samples, to account for small changes in excipient composition. This can be performed quickly on site by the field technician using the instrument software. When calibration needs to be updated with new excipients, previously obtained IR data can simply be reloaded and checked against the new calibration (perhaps performed in a laboratory), without rerunning the original samples. This avoids the necessity of prolonged sample storage which may, by necessity, be in suboptimum conditions. Whereas the present study only concerns one antibiotic, preliminary results on ciprofloxacin and doxycycline show similar agreement between the two techniques (manuscript in preparation). We conclude that the field portable FT-IR may be suitable to intercept substandard antibiotics in developing countries where more sophisticated techniques are not available. The benefits of the FT-IR come from its relatively low cost, its portability, simplicity of use, and rapid analysis time.

## Supplementary Material

Supplemental Text, Tables, and Figures.
