# Supplementary material for: Toward Point-of-Care Drug Quality Assurance in Developing Countries: Comparison of Liquid Chromatography and Infrared Spectroscopy Quantitation of a Small-Scale Random Sample of Amoxicillin
Source: Am J Trop Med Hyg. 2018 Jun 11;99(2):477–81. doi: 10.4269/ajtmh.17-0779 (PMC6090331; doi:10.4269/ajtmh.17-0779)
Supplement: Supplementary file 1 [file tpmd170779.SD1.pdf]

Towards point-of-care drug quality assurance in developing countries: comparison of LC-UV and FT-IR quantitation of a small scale random sample of amoxicillin

Norah Alotaibi,[1] Sean Overton,[1] Sharon Curtis,[1] Jason W. Nickerson,[2, 3] Amir Attaran,[3] Sheldon Gilmer,[4] and Paul M Mayer[1]*

[1]Department of Chemistry and Biomolecular Sciences, University of Ottawa, Canada; [2] Bruyère Research Institute, 85 Primrose Ave., Room 308-B, Ottawa, Ontario, Canada; [3] Faculty of Law, University of Ottawa, Ottawa, Ontario, Canada; [4] World Hope Canada 210 Prescott Street, Suite 3A PO Box 982 Kemptville, ON K0G 1J0 CANADA

*address correspondence to Paul M Mayer, Department of Chemistry and Biomolecular Sciences, University of Ottawa, Canada K1N 6N5 Email:pmmayer@uottawa.ca

Supporting information

Table S1: Sample information, capsule weight analysis, LC-UV results, FT-IR results

Figure S1: FT-IR spectrum of amoxicillin and the combined picture of the calibration set.

Sample information

SAMPLE INFORMATION					SAMPLING INFORMATION		
Sample #	API form	Formulation	Strength		Country	City	Location type
AMX0001	Trihydrate	CAPS	500		DRC	Kinshasa	Central Depot
AMX0002	Trihydrate	CAPS	500		DRC	Kinshasa	Central Depot
AMX0003	Trihydrate	CAPS	500		DRC	Kinshasa	Central Depot
AMX0004	Trihydrate	CAPS	500		DRC	Kinshasa	Central Depot
AMX0005	Trihydrate	CAPS	500		DRC	Kinshasa	Central Depot
AMX0006	Trihydrate	CAPS	250		DRC	Kinshasa	Central Depot
AMX0007	Trihydrate	CAPS	500		DRC	Kinshasa	Central Depot
AMX0008	Trihydrate	CAPS	250		GHA	Accra	Central Depot
AMX0009	Trihydrate	CAPS	250		GHA	Accra	Central Depot
AMX0010	Trihydrate	CAPS	250		GHA	Accra	Central Depot
AMX0011	Trihydrate	CAPS	250		GHA	Accra	Central Depot
AMX0012	Trihydrate	CAPS	500		DRC	Kinshasa	Central Depot
AMX0013	Trihydrate	CAPS	500		DRC	Kinshasa	Central Depot
AMX0023	Trihydrate	CAPS	500		HAI	Port-au-Prince	private pharmacy
AMX0024	Trihydrate	CAPS	500		HAI	Port-au-Prince	private pharmacy
AMX0025	Trihydrate	CAPS	500		IND		private pharmacy
AMX0027	Trihydrate	CAPS	500		PNG		private pharmacy
AMX0028	Trihydrate	CAPS	500		ETH		private pharmacy
AMX0029	Trihydrate	CAPS	500		ETH		private pharmacy
AMX0031	Trihydrate	CAPS	250		SLE	Kamakwie	Hospital pharmacy
AMX0032	Trihydrate	CAPS	500		SLE	Kamakwie	Hospital pharmacy
AMX0033	Trihydrate	CAPS	500		SLE	Makeni	private pharmacy
AMX0034	Trihydrate	CAPS	500		SLE	Makeni	private pharmacy
AMX0035	Trihydrate	CAPS	500		SLE	Makeni	private pharmacy
AMX0036	Trihydrate	CAPS	250		SLE	Makeni	private pharmacy
AMX0037	Trihydrate	CAPS	500		SLE	Makeni	private pharmacy
AMX0038	Trihydrate	CAPS	250		SLE	Makeni	private pharmacy
AMX0039	Trihydrate	CAPS	500		SLE	Freetown	private pharmacy
AMX0040	Trihydrate	CAPS	500		SLE	Freetown	private pharmacy
AMX0041	Trihydrate	CAPS	500		GHA	Tema	private pharmacy
AMX0042	Trihydrate	CAPS	500		GHA	Atimpoku/Volta River	private pharmacy
AMX0043	Trihydrate	CAPS	500		GHA	Tema	private pharmacy
AMX0044	Trihydrate	CAPS	500		GHA	Tema	private pharmacy
AMX0045	Trihydrate	CAPS	500		GHA	Tema	private pharmacy
AMX0046	Trihydrate	CAPS	500		GHA	Tema	private pharmacy
AMX0047	Trihydrate	CAPS	500		GHA	Tema	private pharmacy
AMX0048	Trihydrate	CAPS	500		HAI	Port-au-Prince	Market Stall
AMX0050	Trihydrate	CAPS	250		DRC	Kinshasa	Central Depot
AMX0051	Trihydrate	CAPS	500		DRC	Kinshasa	Central Depot

Capsule mass analyses

Sample #	SAMP. LOC.	Total mass	RMD	AVERAGE	STDEV	STDEV%	Capsule Mass	RMD	AVERAGE	STDEV	STDEV%	Content Mass	RMD	AVERAGE	STDEV	STDEV%
AMXSTD1	CANADA	725.6	0.1%	725	8	1%	103.3	-0.9%	104	2	2%	622.3	0.3%	620.7	8	1%
AMXSTD1	CANADA	723.5	-0.2%	725	8	1%	101.7	-2.4%	104	2	2%	621.8	0.2%	620.7	8	1%
AMXSTD1	CANADA	738.8	1.9%	725	8	1%	106.7	2.4%	104	2	2%	632.1	1.8%	620.7	8	1%
AMXSTD1	CANADA	717.4	-1.0%	725	8	1%	104.1	-0.1%	104	2	2%	613.3	-1.2%	620.7	8	1%
AMXSTD1	CANADA	720.0	-0.7%	725	8	1%	101.6	-2.5%	104	2	2%	618.4	-0.4%	620.7	8	1%
AMXSTD1	CANADA	710.7	-2.0%	725	8	1%	106.2	1.9%	104	2	2%	604.5	-2.6%	620.7	8	1%
AMXSTD1	CANADA	726.4	0.2%	725	8	1%	105.8	1.5%	104	2	2%	620.6	0.0%	620.7	8	1%
AMXSTD1	CANADA	732.7	1.1%	725	8	1%	103.6	-0.6%	104	2	2%	629.1	1.3%	620.7	8	1%
AMXSTD1	CANADA	729.6	0.6%	725	8	1%	105.1	0.8%	104	2	2%	624.5	0.6%	620.7	8	1%
AMX0001	DRC	667.4	2.1%	654	20	3%	103.7	2.8%	101	3	3%	563.7	2.0%	553	17	3%
AMX0001	DRC	670.5	2.6%	654	20	3%	102.9	2.0%	101	3	3%	567.6	2.7%	553	17	3%
AMX0001	DRC	636.1	-2.7%	654	20	3%	100.0	-0.8%	101	3	3%	536.1	-3.0%	553	17	3%
AMX0001	DRC	627.7	-4.0%	654	20	3%	96.2	-4.6%	101	3	3%	531.5	-3.8%	553	17	3%
AMX0001	DRC	665.9	1.9%	654	20	3%	101.4	0.6%	101	3	3%	564.5	2.1%	553	17	3%
AMX0002	DRC	659.5	-0.3%	661	10	2%	100.3	3.0%	97	2	2%	559.2	-0.9%	564	10	2%
AMX0002	DRC	650.3	-1.7%	661	10	2%	96.5	-0.9%	97	2	2%	553.8	-1.8%	564	10	2%
AMX0002	DRC	677.4	2.4%	661	10	2%	98.6	1.3%	97	2	2%	578.8	2.6%	564	10	2%
AMX0002	DRC	664.7	0.5%	661	10	2%	95.4	-2.0%	97	2	2%	569.3	0.9%	564	10	2%
AMX0002	DRC	655.5	-0.9%	661	10	2%	96.0	-1.4%	97	2	2%	559.5	-0.8%	564	10	2%
AMX0003	DRC	672.0	-0.8%	677	7	1%	101.4	5.3%	96	3	3%	570.6	-1.8%	581	9	2%
AMX0003	DRC	680.9	0.5%	677	7	1%	94.6	-1.7%	96	3	3%	586.3	0.9%	581	9	2%
AMX0003	DRC	668.2	-1.3%	677	7	1%	95.6	-0.7%	96	3	3%	572.6	-1.4%	581	9	2%
AMX0003	DRC	687.0	1.4%	677	7	1%	94.3	-2.0%	96	3	3%	592.7	2.0%	581	9	2%
AMX0003	DRC	678.2	0.1%	677	7	1%	95.4	-0.9%	96	3	3%	582.8	0.3%	581	9	2%
AMX0004	DRC	674.2	0.1%	674	10	1%	99.6	-0.4%	100	2	2%	574.6	0.2%	574	12	2%
AMX0004	DRC	679.0	0.8%	674	10	1%	99.3	-0.7%	100	2	2%	579.7	1.1%	574	12	2%
AMX0004	DRC	678.1	0.7%	674	10	1%	97.7	-2.3%	100	2	2%	580.4	1.2%	574	12	2%
AMX0004	DRC	656.8	-2.5%	674	10	1%	104.1	4.1%	100	2	2%	552.7	-3.6%	574	12	2%
AMX0004	DRC	680.0	0.9%	674	10	1%	99.4	-0.6%	100	2	2%	580.6	1.2%	574	12	2%
AMX0005	DRC	710.7	2.0%	697	10	1%	107.8	0.5%	107	2	2%	602.9	2.2%	590	10	2%
AMX0005	DRC	697.0	0.0%	697	10	1%	105.6	-1.5%	107	2	2%	591.4	0.3%	590	10	2%
AMX0005	DRC	687.9	-1.3%	697	10	1%	109.0	1.7%	107	2	2%	578.9	-1.8%	590	10	2%
AMX0005	DRC	686.9	-1.4%	697	10	1%	105.2	-1.9%	107	2	2%	581.7	-1.4%	590	10	2%
AMX0005	DRC	702.3	0.8%	697	10	1%	108.5	1.2%	107	2	2%	593.8	0.7%	590	10	2%
AMX0006	DRC	364.5	-2.4%	374	8	2%	60.6	-2.3%	62	3	5%	303.9	-2.5%	312	8	3%
AMX0006	DRC	375.4	0.5%	374	8	2%	66.7	7.5%	62	3	5%	308.7	-0.9%	312	8	3%
AMX0006	DRC	386.1	3.3%	374	8	2%	60.1	-3.1%	62	3	5%	326.0	4.6%	312	8	3%

capsule mass analysis

AMX0006	DRC	373.2	-0.1%	374	8	2%	63.1	1.7%	62	3	5%	310.1	-0.5%	312	8	3%
AMX0006	DRC	368.8	-1.3%	374	8	2%	59.7	-3.8%	62	3	5%	309.1	-0.8%	312	8	3%
AMX0007	DRC	700.5	1.8%	688	10	1%	96.3	1.3%	95	3	3%	604.2	1.9%	593	9	2%
AMX0007	DRC	695.4	1.0%	688	10	1%	94.2	-0.9%	95	3	3%	601.2	1.3%	593	9	2%
AMX0007	DRC	685.3	-0.4%	688	10	1%	98.5	3.6%	95	3	3%	586.8	-1.1%	593	9	2%
AMX0007	DRC	674.0	-2.1%	688	10	1%	91.8	-3.4%	95	3	3%	582.2	-1.9%	593	9	2%
AMX0007	DRC	686.2	-0.3%	688	10	1%	94.5	-0.6%	95	3	3%	591.7	-0.3%	593	9	2%
AMX0008C	GHA	812.4	1.9%	797	10	1%	154.5	1.0%	153	1	1%	657.9	2.1%	644	9	1%
AMX0008C	GHA	787.3	-1.2%	797	10	1%	151.4	-1.0%	153	1	1%	635.9	-1.3%	644	9	1%
AMX0008C	GHA	797.0	0.0%	797	10	1%	153.5	0.3%	153	1	1%	643.5	-0.1%	644	9	1%
AMX0008C	GHA	798.2	0.1%	797	10	1%	152.6	-0.2%	153	1	1%	645.6	0.2%	644	9	1%
AMX0008C	GHA	791.4	-0.7%	797	10	1%	152.9	-0.1%	153	1	1%	638.5	-0.9%	644	9	1%
AMX0009C	GHA	793.9	-0.3%	796	9	1%	158.4	2.3%	155	3	2%	635.5	-0.9%	641	9	1%
AMX0009C	GHA	785.5	-1.3%	796	9	1%	152.2	-1.7%	155	3	2%	633.3	-1.2%	641	9	1%
AMX0009C	GHA	795.7	0.0%	796	9	1%	152.4	-1.6%	155	3	2%	643.3	0.3%	641	9	1%
AMX0009C	GHA	794.9	-0.1%	796	9	1%	155.8	0.6%	155	3	2%	639.1	-0.3%	641	9	1%
AMX0009C	GHA	810.4	1.8%	796	9	1%	155.5	0.4%	155	3	2%	654.9	2.1%	641	9	1%
AMX0010C	GHA	805.6	0.0%	806	10	1%	153.0	0.1%	153	1	1%	652.6	0.0%	653	10	1%
AMX0010C	GHA	813.8	1.0%	806	10	1%	152.6	-0.2%	153	1	1%	661.2	1.3%	653	10	1%
AMX0010C	GHA	790.1	-1.9%	806	10	1%	153.5	0.4%	153	1	1%	636.6	-2.5%	653	10	1%
AMX0010C	GHA	805.3	0.0%	806	10	1%	151.2	-1.1%	153	1	1%	654.1	0.2%	653	10	1%
AMX0010C	GHA	813.7	1.0%	806	10	1%	154.3	0.9%	153	1	1%	659.4	1.0%	653	10	1%
AMX0011C	GHA	806.4	1.5%	795	7	1%	156.9	0.2%	157	2	1%	649.5	1.8%	638	7	1%
AMX0011C	GHA	792.2	-0.3%	795	7	1%	157.7	0.8%	157	2	1%	634.5	-0.6%	638	7	1%
AMX0011C	GHA	790.3	-0.5%	795	7	1%	157.5	0.6%	157	2	1%	632.8	-0.8%	638	7	1%
AMX0011C	GHA	789.5	-0.6%	795	7	1%	156.8	0.2%	157	2	1%	632.7	-0.8%	638	7	1%
AMX0011C	GHA	794.8	0.0%	795	7	1%	153.7	-1.8%	157	2	1%	641.1	0.5%	638	7	1%
AMX0012	DRC	612.7	-3.5%	635	28	4%	95.8	-0.1%	96	1	1%	516.9	-4.1%	539	29	5%
AMX0012	DRC	648.6	2.2%	635	28	4%	97.7	1.9%	96	1	1%	550.9	2.2%	539	29	5%
AMX0012	DRC	598.9	-5.6%	635	28	4%	96.2	0.4%	96	1	1%	502.7	-6.7%	539	29	5%
AMX0012	DRC	668.1	5.3%	635	28	4%	94.4	-1.5%	96	1	1%	573.7	6.5%	539	29	5%
AMX0012	DRC	645.2	1.7%	635	28	4%	95.2	-0.7%	96	1	1%	550.0	2.1%	539	29	5%
AMX0023A	HAI	707.9	13.3%	625	66	11%	91.0	-0.6%	92	2	2%	616.9	15.7%	533	66	12%
AMX0023A	HAI	670.1	7.3%	625	66	11%	91.7	0.2%	92	2	2%	578.4	8.5%	533	66	12%
AMX0023A	HAI	560.4	-10.3%	625	66	11%	93.1	1.7%	92	2	2%	467.3	-12.3%	533	66	12%
AMX0023A	HAI	557.5	-10.7%	625	66	11%	88.1	-3.7%	92	2	2%	469.4	-11.9%	533	66	12%
AMX0023A	HAI	626.8	0.4%	625	66	11%	93.7	2.4%	92	2	2%	533.1	0.0%	533	66	12%
AMX0023B	HAI	695.9	0.0%	696	16	2%	92.6	1.5%	91	2	2%	603.3	-0.2%	605	17	3%

capsule mass analysis

AMX0023B	HAI	679.5	-2.3%	696	16	2%	91.0	-0.2%	91	2	2%	588.5	-2.7%	605	17	3%
AMX0023B	HAI	710.4	2.1%	696	16	2%	90.9	-0.3%	91	2	2%	619.5	2.5%	605	17	3%
AMX0023B	HAI	681.1	-2.1%	696	16	2%	93.5	2.5%	91	2	2%	587.6	-2.8%	605	17	3%
AMX0023B	HAI	712.3	2.4%	696	16	2%	88.0	-3.5%	91	2	2%	624.3	3.3%	605	17	3%
AMX0024A	HAI	701.4	1.4%	692	30	4%	91.5	0.5%	91	1	1%	609.9	1.5%	601	30	5%
AMX0024A	HAI	702.4	1.5%	692	30	4%	91.2	0.1%	91	1	1%	611.2	1.8%	601	30	5%
AMX0024A	HAI	642.1	-7.2%	692	30	4%	90.2	-1.0%	91	1	1%	551.9	-8.1%	601	30	5%
AMX0024A	HAI	723.3	4.6%	692	30	4%	91.4	0.4%	91	1	1%	631.9	5.2%	601	30	5%
AMX0024A	HAI	689.3	-0.3%	692	30	4%	91.1	0.0%	91	1	1%	598.2	-0.4%	601	30	5%
AMX0024B	HAI	690.5	7.3%	644	95	15%	92.2	0.2%	92	0	0%	598.3	8.4%	552	95	17%
AMX0024B	HAI	475.1	-26.2%	644	95	15%	91.8	-0.3%	92	0	0%	383.3	-30.5%	552	95	17%
AMX0024B	HAI	671.9	4.4%	644	95	15%	92.6	0.6%	92	0	0%	579.3	5.0%	552	95	17%
AMX0024B	HAI	698.0	8.4%	644	95	15%	91.6	-0.5%	92	0	0%	606.4	9.9%	552	95	17%
AMX0024B	HAI	683.5	6.2%	644	95	15%	92.1	0.0%	92	0	0%	591.4	7.2%	552	95	17%
AMX0025	IND	693.7	-0.2%	695	6	1%	94.7	0.9%	94	2	2%	599.0	-0.4%	601	5	1%
AMX0025	IND	695.0	0.0%	695	6	1%	95.2	1.5%	94	2	2%	599.8	-0.2%	601	5	1%
AMX0025	IND	696.6	0.2%	695	6	1%	93.5	-0.3%	94	2	2%	603.1	0.3%	601	5	1%
AMX0025	IND	703.9	1.3%	695	6	1%	94.6	0.8%	94	2	2%	609.3	1.3%	601	5	1%
AMX0025	IND	686.2	-1.3%	695	6	1%	91.1	-2.9%	94	2	2%	595.1	-1.0%	601	5	1%
AMX0027	PNG	731.3	0.7%	726	10	1%	101.9	0.4%	102	2	2%	629.4	0.8%	625	9	1%
AMX0027	PNG	738.2	1.6%	726	10	1%	105.3	3.7%	102	2	2%	632.9	1.3%	625	9	1%
AMX0027	PNG	727.5	0.2%	726	10	1%	100.4	-1.1%	102	2	2%	627.1	0.4%	625	9	1%
AMX0027	PNG	724.0	-0.3%	726	10	1%	100.7	-0.8%	102	2	2%	623.3	-0.2%	625	9	1%
AMX0027	PNG	710.1	-2.2%	726	10	1%	99.4	-2.1%	102	2	2%	610.7	-2.2%	625	9	1%
AMX0028	ETH	707.5	1.0%	701	18	3%	108.4	-2.3%	111	2	2%	599.1	1.6%	590	17	3%
AMX0028	ETH	724.7	3.4%	701	18	3%	114.4	3.2%	111	2	2%	610.3	3.5%	590	17	3%
AMX0028	ETH	676.9	-3.4%	701	18	3%	111.5	0.5%	111	2	2%	565.4	-4.1%	590	17	3%
AMX0028	ETH	701.0	0.1%	701	18	3%	110.0	-0.8%	111	2	2%	591.0	0.2%	590	17	3%
AMX0028	ETH	693.1	-1.1%	701	18	3%	110.2	-0.6%	111	2	2%	582.9	-1.2%	590	17	3%
AMX0029A	ETH	670.8	0.2%	670	2	0%	96.4	-0.8%	97	2	2%	574.4	0.3%	572	2	0%
AMX0029A	ETH	671.3	0.3%	670	2	0%	97.1	-0.1%	97	2	2%	574.2	0.3%	572	2	0%
AMX0029A	ETH	666.1	-0.5%	670	2	0%	95.6	-1.6%	97	2	2%	570.5	-0.3%	572	2	0%
AMX0029A	ETH	668.9	-0.1%	670	2	0%	96.6	-0.6%	97	2	2%	572.3	0.0%	572	2	0%
AMX0029A	ETH	670.9	0.2%	670	2	0%	100.1	3.0%	97	2	2%	570.8	-0.3%	572	2	0%
AMX0029B	ETH	670.3	-0.2%	672	1	0%	97.2	1.2%	96	2	2%	573.1	-0.5%	576	2	0%
AMX0029B	ETH	673.2	0.2%	672	1	0%	97.6	1.6%	96	2	2%	575.6	0.0%	576	2	0%
AMX0029B	ETH	670.7	-0.2%	672	1	0%	92.3	-3.9%	96	2	2%	578.4	0.4%	576	2	0%
AMX0029B	ETH	673.5	0.2%	672	1	0%	96.5	0.4%	96	2	2%	577.0	0.2%	576	2	0%

capsule mass analysis

AMX0029B	ETH	672.1	0.0%	672	1	0%	96.8	0.7%	96	2	2%	575.3	-0.1%	576	2	0%
AMX0031A	SLE	385.8	-1.9%	393	5	1%	80.9	0.8%	80	2	2%	304.9	-2.6%	313	5	2%
AMX0031A	SLE	393.4	0.0%	393	5	1%	80.6	0.4%	80	2	2%	312.8	-0.1%	313	5	2%
AMX0031A	SLE	393.8	0.1%	393	5	1%	81.5	1.5%	80	2	2%	312.3	-0.2%	313	5	2%
AMX0031A	SLE	394.9	0.4%	393	5	1%	76.8	-4.3%	80	2	2%	318.1	1.6%	313	5	2%
AMX0031A	SLE	398.7	1.4%	393	5	1%	81.5	1.5%	80	2	2%	317.2	1.3%	313	5	2%
AMX0031B	SLE	397.3	1.3%	392	7	2%	78.2	0.3%	78	3	3%	319.1	1.5%	314	7	2%
AMX0031B	SLE	398.7	1.6%	392	7	2%	75.9	-2.7%	78	3	3%	322.8	2.7%	314	7	2%
AMX0031B	SLE	381.6	-2.7%	392	7	2%	75.1	-3.7%	78	3	3%	306.5	-2.5%	314	7	2%
AMX0031B	SLE	394.9	0.7%	392	7	2%	81.1	4.0%	78	3	3%	313.8	-0.2%	314	7	2%
AMX0031B	SLE	389.1	-0.8%	392	7	2%	79.6	2.1%	78	3	3%	309.5	-1.5%	314	7	2%
AMX0031C	SLE	392.7	0.5%	391	3	1%	76.8	-5.0%	81	6	7%	315.9	2.0%	310	5	2%
AMX0031C	SLE	388.6	-0.5%	391	3	1%	77.9	-3.7%	81	6	7%	310.7	0.3%	310	5	2%
AMX0031C	SLE	387.0	-0.9%	391	3	1%	80.9	0.0%	81	6	7%	306.1	-1.2%	310	5	2%
AMX0031C	SLE	393.4	0.7%	391	3	1%	90.4	11.8%	81	6	7%	303.0	-2.2%	310	5	2%
AMX0031C	SLE	391.1	0.1%	391	3	1%	78.3	-3.2%	81	6	7%	312.8	1.0%	310	5	2%
AMX0032	SLE	688.8	-0.7%	694	8	1%	101.2	-4.4%	106	3	2%	587.6	-0.1%	588	7	1%
AMX0032	SLE	684.0	-1.4%	694	8	1%	107.0	1.1%	106	3	2%	577.0	-1.9%	588	7	1%
AMX0032	SLE	698.0	0.6%	694	8	1%	106.7	0.8%	106	3	2%	591.3	0.6%	588	7	1%
AMX0032	SLE	704.8	1.6%	694	8	1%	107.6	1.6%	106	3	2%	597.2	1.6%	588	7	1%
AMX0032	SLE	694.1	0.0%	694	8	1%	106.9	1.0%	106	3	2%	587.2	-0.1%	588	7	1%
AMX0033A	SLE	725.7	-0.3%	728	3	0%	127.0	-0.7%	128	2	1%	598.7	-0.2%	600	4	1%
AMX0033A	SLE	729.7	0.3%	728	3	0%	127.8	0.0%	128	2	1%	601.9	0.4%	600	4	1%
AMX0033A	SLE	723.7	-0.5%	728	3	0%	130.1	1.8%	128	2	1%	593.6	-1.0%	600	4	1%
AMX0033A	SLE	730.7	0.4%	728	3	0%	125.6	-1.8%	128	2	1%	605.1	0.9%	600	4	1%
AMX0033A	SLE	727.8	0.0%	728	3	0%	128.7	0.7%	128	2	1%	599.1	-0.1%	600	4	1%
AMX0033B	SLE	729.3	-0.8%	735	8	1%	128.0	-0.4%	128	2	2%	601.3	-0.9%	607	9	1%
AMX0033B	SLE	734.9	-0.1%	735	8	1%	131.5	2.4%	128	2	2%	603.4	-0.6%	607	9	1%
AMX0033B	SLE	744.3	1.2%	735	8	1%	129.0	0.4%	128	2	2%	615.3	1.4%	607	9	1%
AMX0033B	SLE	725.4	-1.4%	735	8	1%	128.3	-0.1%	128	2	2%	597.1	-1.6%	607	9	1%
AMX0033B	SLE	743.1	1.0%	735	8	1%	125.5	-2.3%	128	2	2%	617.6	1.8%	607	9	1%
AMX0033C	SLE	720.7	-1.0%	728	9	1%	126.2	-0.1%	126	2	1%	594.5	-1.1%	601	8	1%
AMX0033C	SLE	716.2	-1.6%	728	9	1%	125.3	-0.8%	126	2	1%	590.9	-1.7%	601	8	1%
AMX0033C	SLE	736.8	1.3%	728	9	1%	129.2	2.3%	126	2	1%	607.6	1.0%	601	8	1%
AMX0033C	SLE	732.5	0.7%	728	9	1%	124.6	-1.3%	126	2	1%	607.9	1.1%	601	8	1%
AMX0033C	SLE	732.0	0.6%	728	9	1%	126.1	-0.1%	126	2	1%	605.9	0.8%	601	8	1%
AMX0033D	SLE	736.4	0.3%	734	4	1%	124.6	-2.5%	128	2	2%	611.8	1.0%	606	6	1%
AMX0033D	SLE	727.5	-0.9%	734	4	1%	129.9	1.6%	128	2	2%	597.6	-1.4%	606	6	1%

capsule mass analysis

AMX0033D	SLE	736.9	0.4%	734	4	1%	130.3	2.0%	128	2	2%	606.6	0.1%	606	6	1%
AMX0033D	SLE	736.8	0.4%	734	4	1%	126.7	-0.9%	128	2	2%	610.1	0.7%	606	6	1%
AMX0033D	SLE	731.6	-0.3%	734	4	1%	127.5	-0.2%	128	2	2%	604.1	-0.3%	606	6	1%
AMX0034A	SLE	704.7	-1.3%	714	6	1%	107.2	-3.5%	111	2	2%	597.5	-0.9%	603	5	1%
AMX0034A	SLE	714.3	0.1%	714	6	1%	112.0	0.8%	111	2	2%	602.3	-0.1%	603	5	1%
AMX0034A	SLE	714.0	0.0%	714	6	1%	112.8	1.6%	111	2	2%	601.2	-0.2%	603	5	1%
AMX0034A	SLE	721.8	1.1%	714	6	1%	111.1	0.0%	111	2	2%	610.7	1.3%	603	5	1%
AMX0034A	SLE	713.7	0.0%	714	6	1%	112.2	1.0%	111	2	2%	601.5	-0.2%	603	5	1%
AMX0034B	SLE	710.5	0.2%	709	4	1%	111.2	0.0%	111	2	1%	599.3	0.3%	598	5	1%
AMX0034B	SLE	709.3	0.0%	709	4	1%	109.3	-1.7%	111	2	1%	600.0	0.4%	598	5	1%
AMX0034B	SLE	709.6	0.1%	709	4	1%	110.1	-1.0%	111	2	1%	599.5	0.3%	598	5	1%
AMX0034B	SLE	701.7	-1.0%	709	4	1%	113.1	1.7%	111	2	1%	588.6	-1.5%	598	5	1%
AMX0034B	SLE	713.8	0.7%	709	4	1%	112.3	1.0%	111	2	1%	601.5	0.6%	598	5	1%
AMX0035A	SLE	677.0	-0.9%	683	15	2%	101.6	1.2%	100	3	3%	575.4	-1.3%	583	14	2%
AMX0035A	SLE	680.2	-0.4%	683	15	2%	99.8	-0.6%	100	3	3%	580.4	-0.4%	583	14	2%
AMX0035A	SLE	662.9	-3.0%	683	15	2%	97.7	-2.7%	100	3	3%	565.2	-3.0%	583	14	2%
AMX0035A	SLE	703.6	3.0%	683	15	2%	104.8	4.4%	100	3	3%	598.8	2.8%	583	14	2%
AMX0035A	SLE	691.8	1.3%	683	15	2%	97.9	-2.5%	100	3	3%	593.9	1.9%	583	14	2%
AMX0035B	SLE	674.9	-0.3%	677	13	2%	105.5	5.9%	100	5	5%	569.4	-1.4%	577	11	2%
AMX0035B	SLE	678.1	0.2%	677	13	2%	103.7	4.1%	100	5	5%	574.4	-0.5%	577	11	2%
AMX0035B	SLE	697.0	3.0%	677	13	2%	100.0	0.4%	100	5	5%	597.0	3.4%	577	11	2%
AMX0035B	SLE	672.0	-0.7%	677	13	2%	95.5	-4.1%	100	5	5%	576.5	-0.1%	577	11	2%
AMX0035B	SLE	662.5	-2.1%	677	13	2%	93.3	-6.3%	100	5	5%	569.2	-1.4%	577	11	2%
AMX0036A	SLE	362.4	3.4%	350	9	3%	65.4	4.3%	63	2	4%	297.0	3.2%	288	7	2%
AMX0036A	SLE	354.2	1.1%	350	9	3%	64.8	3.3%	63	2	4%	289.4	0.6%	288	7	2%
AMX0036A	SLE	352.7	0.6%	350	9	3%	62.4	-0.5%	63	2	4%	290.3	0.9%	288	7	2%
AMX0036A	SLE	338.7	-3.4%	350	9	3%	59.9	-4.5%	63	2	4%	278.8	-3.1%	288	7	2%
AMX0036A	SLE	344.3	-1.8%	350	9	3%	61.1	-2.6%	63	2	4%	283.2	-1.6%	288	7	2%
AMX0036B	SLE	315.0	-11.1%	354	25	7%	64.2	-0.1%	64	1	2%	250.8	-13.5%	290	25	9%
AMX0036B	SLE	353.3	-0.3%	354	25	7%	62.7	-2.4%	64	1	2%	290.6	0.2%	290	25	9%
AMX0036B	SLE	373.5	5.4%	354	25	7%	64.8	0.9%	64	1	2%	308.7	6.5%	290	25	9%
AMX0036B	SLE	352.4	-0.5%	354	25	7%	65.9	2.6%	64	1	2%	286.5	-1.2%	290	25	9%
AMX0036B	SLE	376.8	6.4%	354	25	7%	63.6	-1.0%	64	1	2%	313.2	8.0%	290	25	9%
AMX0037A	SLE	675.8	-1.1%	683	22	3%	96.1	-1.2%	97	5	6%	579.7	-1.0%	586	24	4%
AMX0037A	SLE	648.9	-5.0%	683	22	3%	100.8	3.7%	97	5	6%	548.1	-6.4%	586	24	4%
AMX0037A	SLE	708.1	3.7%	683	22	3%	99.7	2.6%	97	5	6%	608.4	3.9%	586	24	4%
AMX0037A	SLE	690.5	1.1%	683	22	3%	101.2	4.1%	97	5	6%	589.3	0.6%	586	24	4%
AMX0037A	SLE	691.8	1.3%	683	22	3%	88.3	-9.2%	97	5	6%	603.5	3.0%	586	24	4%

capsule mass analysis

AMX0038A	SLE	370.6	0.1%	370	3	1%	82.3	1.6%	81	3	3%	288.3	-0.4%	289	3	1%
AMX0038A	SLE	373.0	0.7%	370	3	1%	79.8	-1.5%	81	3	3%	293.2	1.3%	289	3	1%
AMX0038A	SLE	365.9	-1.2%	370	3	1%	77.2	-4.7%	81	3	3%	288.7	-0.2%	289	3	1%
AMX0038A	SLE	370.3	0.0%	370	3	1%	83.9	3.6%	81	3	3%	286.4	-1.0%	289	3	1%
AMX0038A	SLE	371.9	0.4%	370	3	1%	81.9	1.1%	81	3	3%	290.0	0.2%	289	3	1%
AMX0038B	SLE	370.1	0.6%	368	2	1%	82.2	0.9%	81	1	2%	287.9	0.5%	286	2	1%
AMX0038B	SLE	369.5	0.4%	368	2	1%	81.0	-0.6%	81	1	2%	288.5	0.7%	286	2	1%
AMX0038B	SLE	365.8	-0.6%	368	2	1%	79.7	-2.2%	81	1	2%	286.1	-0.1%	286	2	1%
AMX0038B	SLE	367.7	-0.1%	368	2	1%	81.3	-0.2%	81	1	2%	286.4	0.0%	286	2	1%
AMX0038B	SLE	366.5	-0.4%	368	2	1%	83.1	2.0%	81	1	2%	283.4	-1.1%	286	2	1%
AMX0039A	SLE	742.9	1.7%	730	9	1%	126.6	-0.8%	128	2	1%	616.3	2.2%	603	9	1%
AMX0039A	SLE	724.4	-0.8%	730	9	1%	128.1	0.4%	128	2	1%	596.3	-1.1%	603	9	1%
AMX0039A	SLE	722.0	-1.2%	730	9	1%	125.6	-1.6%	128	2	1%	596.4	-1.1%	603	9	1%
AMX0039A	SLE	728.0	-0.3%	730	9	1%	129.6	1.6%	128	2	1%	598.4	-0.7%	603	9	1%
AMX0039A	SLE	735.0	0.6%	730	9	1%	128.1	0.4%	128	2	1%	606.9	0.7%	603	9	1%
AMX0039B	SLE	741.6	0.5%	738	9	1%	130.2	1.1%	129	1	1%	611.4	0.4%	609	10	2%
AMX0039B	SLE	731.1	-0.9%	738	9	1%	129.1	0.2%	129	1	1%	602.0	-1.1%	609	10	2%
AMX0039B	SLE	725.3	-1.7%	738	9	1%	129.2	0.3%	129	1	1%	596.1	-2.1%	609	10	2%
AMX0039B	SLE	746.9	1.2%	738	9	1%	126.5	-1.8%	129	1	1%	620.4	1.9%	609	10	2%
AMX0039B	SLE	743.6	0.8%	738	9	1%	129.1	0.2%	129	1	1%	614.5	0.9%	609	10	2%
AMX0040A	SLE	693.2	-0.2%	695	7	1%	94.8	-1.4%	96	2	2%	598.4	0.0%	599	6	1%
AMX0040A	SLE	687.0	-1.1%	695	7	1%	96.3	0.2%	96	2	2%	590.7	-1.3%	599	6	1%
AMX0040A	SLE	690.1	-0.7%	695	7	1%	94.8	-1.4%	96	2	2%	595.3	-0.5%	599	6	1%
AMX0040A	SLE	700.7	0.9%	695	7	1%	100.1	4.1%	96	2	2%	600.6	0.3%	599	6	1%
AMX0040A	SLE	702.4	1.1%	695	7	1%	94.7	-1.5%	96	2	2%	607.7	1.5%	599	6	1%
AMX0040B	SLE	673.5	-1.9%	686	11	2%	94.6	-0.5%	95	5	5%	578.9	-2.1%	591	8	1%
AMX0040B	SLE	691.8	0.8%	686	11	2%	100.3	5.5%	95	5	5%	591.5	0.0%	591	8	1%
AMX0040B	SLE	693.5	1.1%	686	11	2%	95.8	0.8%	95	5	5%	597.7	1.1%	591	8	1%
AMX0040B	SLE	675.4	-1.6%	686	11	2%	87.4	-8.0%	95	5	5%	588.0	-0.5%	591	8	1%
AMX0040B	SLE	697.2	1.6%	686	11	2%	97.1	2.2%	95	5	5%	600.1	1.5%	591	8	1%
AMX0041	GHA	698.6	0.8%	693	6	1%	97.3	-0.2%	98	1	1%	601.3	1.0%	595	6	1%
AMX0041	GHA	690.5	-0.4%	693	6	1%	97.9	0.4%	98	1	1%	592.6	-0.5%	595	6	1%
AMX0041	GHA	685.6	-1.1%	693	6	1%	96.5	-1.1%	98	1	1%	589.1	-1.1%	595	6	1%
AMX0041	GHA	689.6	-0.5%	693	6	1%	98.5	1.0%	98	1	1%	591.1	-0.7%	595	6	1%
AMX0041	GHA	700.5	1.1%	693	6	1%	97.5	0.0%	98	1	1%	603.0	1.3%	595	6	1%
AMX0042	GHA	699.7	-0.7%	704	4	1%	112.9	0.2%	113	2	2%	586.8	-0.8%	592	3	1%
AMX0042	GHA	709.9	0.8%	704	4	1%	114.5	1.7%	113	2	2%	595.4	0.6%	592	3	1%
AMX0042	GHA	701.7	-0.4%	704	4	1%	109.7	-2.6%	113	2	2%	592.0	0.0%	592	3	1%

capsule mass analysis

AMX0042	GHA	704.9	0.1%	704	4	1%	112.5	-0.1%	113	2	2%	592.4	0.1%	592	3	1%
AMX0042	GHA	705.6	0.2%	704	4	1%	113.5	0.8%	113	2	2%	592.1	0.1%	592	3	1%
AMX0043	GHA	696.6	-1.4%	707	7	1%	111.4	-3.0%	115	3	3%	585.2	-1.1%	592	6	1%
AMX0043	GHA	715.2	1.2%	707	7	1%	113.8	-0.9%	115	3	3%	601.4	1.6%	592	6	1%
AMX0043	GHA	705.9	-0.1%	707	7	1%	114.3	-0.5%	115	3	3%	591.6	0.0%	592	6	1%
AMX0043	GHA	705.9	-0.1%	707	7	1%	114.3	-0.5%	115	3	3%	591.6	0.0%	592	6	1%
AMX0043	GHA	709.4	0.4%	707	7	1%	120.6	5.0%	115	3	3%	588.8	-0.5%	592	6	1%
AMX0044	GHA	699.4	0.8%	694	4	1%	96.8	1.7%	95	1	1%	602.6	0.6%	599	3	1%
AMX0044	GHA	692.8	-0.2%	694	4	1%	94.4	-0.9%	95	1	1%	598.4	-0.1%	599	3	1%
AMX0044	GHA	688.6	-0.8%	694	4	1%	94.1	-1.2%	95	1	1%	594.5	-0.7%	599	3	1%
AMX0044	GHA	692.8	-0.2%	694	4	1%	96.1	0.9%	95	1	1%	596.7	-0.3%	599	3	1%
AMX0044	GHA	696.2	0.3%	694	4	1%	94.7	-0.5%	95	1	1%	601.5	0.5%	599	3	1%
AMX0045	GHA	677.0	2.9%	658	15	2%	96.4	0.5%	96	1	1%	580.6	3.3%	562	14	3%
AMX0045	GHA	655.2	-0.4%	658	15	2%	95.2	-0.8%	96	1	1%	560.0	-0.4%	562	14	3%
AMX0045	GHA	669.9	1.8%	658	15	2%	97.3	1.4%	96	1	1%	572.6	1.9%	562	14	3%
AMX0045	GHA	644.0	-2.1%	658	15	2%	95.1	-0.9%	96	1	1%	548.9	-2.3%	562	14	3%
AMX0045	GHA	643.5	-2.2%	658	15	2%	95.7	-0.3%	96	1	1%	547.8	-2.5%	562	14	3%
AMX0046	GHA	718.7	-1.0%	726	13	2%	97.5	0.7%	97	3	3%	621.2	-1.2%	629	13	2%
AMX0046	GHA	714.2	-1.6%	726	13	2%	99.8	3.0%	97	3	3%	614.4	-2.3%	629	13	2%
AMX0046	GHA	737.7	1.7%	726	13	2%	98.4	1.6%	97	3	3%	639.3	1.7%	629	13	2%
AMX0046	GHA	715.6	-1.4%	726	13	2%	92.5	-4.5%	97	3	3%	623.1	-0.9%	629	13	2%
AMX0046	GHA	742.2	2.3%	726	13	2%	96.1	-0.8%	97	3	3%	646.1	2.7%	629	13	2%
AMX0047	GHA	647.0	-4.9%	680	19	3%	95.5	1.1%	95	2	2%	551.5	-5.8%	586	20	3%
AMX0047	GHA	683.2	0.4%	680	19	3%	96.5	2.1%	95	2	2%	586.7	0.2%	586	20	3%
AMX0047	GHA	693.5	1.9%	680	19	3%	93.3	-1.3%	95	2	2%	600.2	2.5%	586	20	3%
AMX0047	GHA	688.8	1.3%	680	19	3%	92.4	-2.2%	95	2	2%	596.4	1.8%	586	20	3%
AMX0047	GHA	688.8	1.3%	680	19	3%	94.8	0.3%	95	2	2%	594.0	1.4%	586	20	3%
AMX0048A	HAI	682.8	-1.3%	691	12	2%	101.8	3.0%	99	3	3%	581.0	-2.0%	593	12	2%
AMX0048A	HAI	678.2	-1.9%	691	12	2%	98.9	0.0%	99	3	3%	579.3	-2.2%	593	12	2%
AMX0048A	HAI	706.8	2.2%	691	12	2%	100.4	1.6%	99	3	3%	606.4	2.3%	593	12	2%
AMX0048A	HAI	701.4	1.4%	691	12	2%	98.2	-0.7%	99	3	3%	603.2	1.8%	593	12	2%
AMX0048A	HAI	688.1	-0.5%	691	12	2%	95.0	-3.9%	99	3	3%	593.1	0.1%	593	12	2%
AMX0048B	HAI	688.0	1.1%	680	17	3%	101.8	3.7%	98	3	3%	586.2	0.7%	582	15	3%
AMX0048B	HAI	702.9	3.3%	680	17	3%	99.9	1.8%	98	3	3%	603.0	3.6%	582	15	3%
AMX0048B	HAI	656.7	-3.5%	680	17	3%	95.2	-3.0%	98	3	3%	561.5	-3.5%	582	15	3%
AMX0048B	HAI	671.4	-1.3%	680	17	3%	95.7	-2.5%	98	3	3%	575.7	-1.1%	582	15	3%
AMX0048B	HAI	682.2	0.3%	680	17	3%	98.2	0.0%	98	3	3%	584.0	0.3%	582	15	3%
AMX0048C	HAI	680.3	-1.2%	689	7	1%	95.5	-3.9%	99	2	2%	584.8	-0.8%	589	6	1%

capsule mass analysis

AMX0048C	HAI	688.1	-0.1%	689	7	1%	102.0	2.6%	99	2	2%	586.1	-0.5%	589	6	1%
AMX0048C	HAI	698.3	1.4%	689	7	1%	99.9	0.5%	99	2	2%	598.4	1.5%	589	6	1%
AMX0048C	HAI	684.4	-0.6%	689	7	1%	99.5	0.1%	99	2	2%	584.9	-0.8%	589	6	1%
AMX0048C	HAI	692.4	0.5%	689	7	1%	100.0	0.6%	99	2	2%	592.4	0.5%	589	6	1%
AMX0050A	DRC	637.1	-0.5%	640	21	3%	105.1	0.9%	104	2	2%	532.0	-0.8%	536	22	4%
AMX0050A	DRC	606.5	-5.3%	640	21	3%	105.9	1.6%	104	2	2%	500.6	-6.6%	536	22	4%
AMX0050A	DRC	655.0	2.3%	640	21	3%	101.3	-2.8%	104	2	2%	553.7	3.3%	536	22	4%
AMX0050A	DRC	659.7	3.0%	640	21	3%	106.2	1.9%	104	2	2%	553.5	3.3%	536	22	4%
AMX0050A	DRC	643.0	0.4%	640	21	3%	102.5	-1.6%	104	2	2%	540.5	0.8%	536	22	4%
AMX0050B	DRC	609.4	-4.0%	635	21	3%	104.8	2.3%	102	2	2%	504.6	-5.2%	532	22	4%
AMX0050B	DRC	621.1	-2.1%	635	21	3%	100.7	-1.7%	102	2	2%	520.4	-2.2%	532	22	4%
AMX0050B	DRC	660.3	4.1%	635	21	3%	99.7	-2.7%	102	2	2%	560.6	5.4%	532	22	4%
AMX0050B	DRC	650.6	2.5%	635	21	3%	102.4	-0.1%	102	2	2%	548.2	3.0%	532	22	4%
AMX0050B	DRC	631.1	-0.5%	635	21	3%	104.8	2.3%	102	2	2%	526.3	-1.1%	532	22	4%
AMX0051A	DRC	699.0	0.1%	698	1	0%	93.1	-1.1%	94	1	1%	605.9	0.3%	604	2	0%
AMX0051A	DRC	697.2	-0.1%	698	1	0%	93.5	-0.7%	94	1	1%	603.7	-0.1%	604	2	0%
AMX0051A	DRC	698.8	0.1%	698	1	0%	95.0	0.9%	94	1	1%	603.8	0.0%	604	2	0%
AMX0051A	DRC	697.1	-0.2%	698	1	0%	95.4	1.3%	94	1	1%	601.7	-0.4%	604	2	0%
AMX0051A	DRC	699.0	0.1%	698	1	0%	93.8	-0.4%	94	1	1%	605.2	0.2%	604	2	0%
AMX0051B	DRC	698.0	-0.4%	701	4	1%	94.6	-2.1%	97	2	2%	603.4	-0.2%	604	4	1%
AMX0051B	DRC	701.0	0.0%	701	4	1%	98.0	1.4%	97	2	2%	603.0	-0.2%	604	4	1%
AMX0051B	DRC	705.7	0.7%	701	4	1%	100.0	3.5%	97	2	2%	605.7	0.2%	604	4	1%
AMX0051B	DRC	695.5	-0.8%	701	4	1%	95.8	-0.9%	97	2	2%	599.7	-0.8%	604	4	1%
AMX0051B	DRC	705.3	0.6%	701	4	1%	94.9	-1.8%	97	2	2%	610.4	1.0%	604	4	1%

UPLC-UV Analyses

Sample #	SAMP. LOC.	%w/w			RMD	AVERAGE	STDEV	STDEV%	API mass			RMD	AVERAGE	STDEV	STDEV%	%API		
AMXSTD1	CANADA	79.1%	±	0.7%	-1.9%	1	0	4%	492	±	5	-1.7%	501	23	5%	98.4%	±	0.9%
AMXSTD1	CANADA	78.9%	±	1.3%	-2.2%	1	0	4%	491	±	6	-2.0%	501	23	5%	98.1%	±	1.1%
AMXSTD1	CANADA	76.7%	±	6.0%	-4.9%	1	0	4%	485	±	7	-3.2%	501	23	5%	97.0%	±	1.3%
AMXSTD1	CANADA	77.0%	±	0.8%	-4.5%	1	0	4%	472	±	8	-5.6%	501	23	5%	94.5%	±	1.5%
AMXSTD1	CANADA	81.1%	±	3.3%	0.5%	1	0	4%	501	±	9	0.1%	501	23	5%	100.3%	±	1.7%
AMXSTD1	CANADA	82.3%	±	0.8%	2.0%	1	0	4%	497	±	10	-0.7%	501	23	5%	99.4%	±	1.9%
AMXSTD1	CANADA	79.5%	±	8.2%	-1.4%	1	0	4%	493	±	11	-1.5%	501	23	5%	98.7%	±	2.1%
AMXSTD1	CANADA	87.0%	±	1.6%	7.9%	1	0	4%	547	±	12	9.3%	501	23	5%	109.4%	±	2.3%
AMXSTD1	CANADA	84.3%	±	0.1%	4.5%	1	0	4%	527	±	13	5.2%	501	23	5%	105.3%	±	2.5%
AMX0001	DRC	89.9%	±	0.7%	2.3%	88%	1%	2%	507	±	4	4.3%	486	16	3%	101.4%	±	0.8%
AMX0001	DRC	88%	±	2%	-0.5%	88%	1%	2%	497	±	10	2.2%	486	16	3%	99.3%	±	2.0%
AMX0001	DRC	87.7%	±	0.5%	-0.3%	87.9%	1%	2%	470	±	3	-3.2%	486	16	3%	94.0%	±	0.5%
AMX0001	DRC	88%	±	3%	0.5%	88%	1%	2%	469	±	15	-3.4%	486	16	3%	93.9%	±	2.9%
AMX0001	DRC	86.1%	±	0.2%	-2.0%	88%	1%	2%	486	±	1	0.1%	486	16	3%	97.2%	±	0.2%
AMX0002	DRC	89.2%	±	0.3%	1.5%	1	0	1%	499	±	2	0.6%	495	13	3%	99.7%	±	0.3%
AMX0002	DRC	86.9%	±	0.4%	-1.1%	1	0	1%	481	±	2	-2.9%	495	13	3%	96.2%	±	0.5%
AMX0002	DRC	88.6%	±	0.0%	0.9%	1	0	1%	513	±	0	3.5%	495	13	3%	102.6%	±	0.0%
AMX0002	DRC	88.0%	±	0.9%	0.3%	1	0	1%	501	±	5	1.2%	495	13	3%	100.2%	±	1.0%
AMX0002	DRC	86.4%	±	0.1%	-1.6%	1	0	1%	484	±	0	-2.4%	495	13	3%	96.7%	±	0.1%
AMX0003	DRC	84.9%	±	0.1%	-2.7%	1	0	2%	484	±	1	-4.5%	507	14	3%	96.9%	±	0.1%
AMX0003	DRC	87.4%	±	1.0%	0.1%	1	0	2%	512	±	6	1.0%	507	14	3%	102.5%	±	1.2%
AMX0003	DRC	88.7%	±	0.7%	1.6%	1	0	2%	508	±	4	0.1%	507	14	3%	101.5%	±	0.8%
AMX0003	DRC	85.6%	±	2.7%	-2.0%	1	0	2%	507	±	16	0.0%	507	14	3%	101.4%	±	3.1%
AMX0003	DRC	89.8%	±	2.1%	2.9%	1	0	2%	523	±	12	3.2%	507	14	3%	104.7%	±	2.4%
AMX0004	DRC	85.5%	±	0.5%	-0.6%	1	0	2%	491	±	3	-0.4%	493	4	1%	98.3%	±	0.6%
AMX0004	DRC	84.7%	±	0.6%	-1.6%	1	0	2%	491	±	3	-0.5%	493	4	1%	98.2%	±	0.7%
AMX0004	DRC	85.4%	±	0.2%	-0.7%	1	0	2%	495	±	1	0.5%	493	4	1%	99.1%	±	0.2%
AMX0004	DRC	88.4%	±	2.2%	2.8%	1	0	2%	489	±	12	-0.9%	493	4	1%	97.7%	±	2.4%
AMX0004	DRC	86.0%	±	1.0%	0.1%	1	0	2%	500	±	6	1.3%	493	4	1%	99.9%	±	1.2%
AMX0005	DRC	88.4%	±	0.7%	0.6%	1	0	1%	533	±	4	2.9%	518	11	2%	106.6%	±	0.8%
AMX0005	DRC	89.0%	±	0.5%	1.3%	1	0	1%	526	±	3	1.6%	518	11	2%	105.3%	±	0.6%
AMX0005	DRC	87.9%	±	0.2%	0.0%	1	0	1%	509	±	1	-1.8%	518	11	2%	101.7%	±	0.2%
AMX0005	DRC	88.0%	±	2.2%	0.2%	1	0	1%	512	±	13	-1.2%	518	11	2%	102.4%	±	2.6%

UPLC-UV analysis

AMX0005	DRC	86.0%	±	0.7%	-2.1%	1	0	1%	511	±	4	-1.4%	518	11	2%	102.1%	±	0.9%
AMX0006	DRC	82.5%	±	1.8%	0.9%	1	0	2%	251	±	5	-1.5%	255	6	2%	100.3%	±	2.2%
AMX0006	DRC	82.1%	±	2.2%	0.4%	1	0	2%	253	±	7	-0.5%	255	6	2%	101.4%	±	2.7%
AMX0006	DRC	80.6%	±	5.5%	-1.4%	1	0	2%	263	±	18	3.2%	255	6	2%	105.1%	±	7.2%
AMX0006	DRC	83.2%	±	0.7%	1.8%	1	0	2%	258	±	2	1.3%	255	6	2%	103.2%	±	0.8%
AMX0006	DRC	80.3%	±	0.8%	-1.8%	1	0	2%	248	±	3	-2.5%	255	6	2%	99.3%	±	1.0%
AMX0007	DRC	83.3%	±	5.5%	5.2%	1	0	3%	504	±	33	7.2%	470	22	5%	100.7%	±	6.7%
AMX0007	DRC	79.8%	±	4.2%	0.8%	1	0	3%	480	±	25	2.1%	470	22	5%	96.0%	±	5.0%
AMX0007	DRC	79.1%	±	3.2%	-0.1%	1	0	3%	464	±	19	-1.3%	470	22	5%	92.8%	±	3.7%
AMX0007	DRC	76.9%	±	0.6%	-2.9%	1	0	3%	448	±	3	-4.7%	470	22	5%	89.5%	±	0.6%
AMX0007	DRC	76.8%	±	2.4%	-3.0%	1	0	3%	454	±	14	-3.3%	470	22	5%	90.9%	±	2.9%
AMX0008C	GHA	76.4%	±	0.2%	2.2%	1	0	3%	251	±	1	4.4%	241	10	4%	100.5%	±	0.3%
AMX0008C	GHA	76.0%	±	0.0%	1.8%	1	0	3%	242	±	0	0.4%	241	10	4%	96.7%	±	0.0%
AMX0008C	GHA	76.6%	±	0.5%	2.5%	1	0	3%	247	±	2	2.4%	241	10	4%	98.6%	±	0.7%
AMX0008C	GHA	74.2%	±	0.1%	-0.7%	1	0	3%	239	±	0	-0.5%	241	10	4%	95.8%	±	0.1%
AMX0008C	GHA	70.4%	±	3.5%	-5.8%	1	0	3%	225	±	11	-6.6%	241	10	4%	89.9%	±	4.5%
AMX0009C	GHA	67.6%	±	3.8%	-13.4%	1	0	8%	215	±	12	-14.2%	250	21	8%	85.9%	±	4.9%
AMX0009C	GHA	81.3%	±	0.7%	4.3%	1	0	8%	258	±	2	2.9%	250	21	8%	103.0%	±	0.8%
AMX0009C	GHA	77.6%	±	4.2%	-0.6%	1	0	8%	249	±	14	-0.3%	250	21	8%	99.8%	±	5.4%
AMX0009C	GHA	82.8%	±	5.0%	6.1%	1	0	8%	265	±	16	5.7%	250	21	8%	105.8%	±	6.4%
AMX0009C	GHA	80.9%	±	1.3%	3.7%	1	0	8%	265	±	4	5.8%	250	21	8%	105.9%	±	1.7%
AMX0010C	GHA	80.5%	±	2.3%	4.4%	1	0	3%	263	±	7	4.4%	252	8	3%	105.1%	±	2.9%
AMX0010C	GHA	76.0%	±	0.7%	-1.4%	1	0	3%	251	±	2	-0.1%	252	8	3%	100.5%	±	0.9%
AMX0010C	GHA	75.5%	±	0.4%	-2.0%	1	0	3%	240	±	1	-4.5%	252	8	3%	96.2%	±	0.6%
AMX0010C	GHA	76.3%	±	1.0%	-1.1%	1	0	3%	249	±	3	-0.9%	252	8	3%	99.8%	±	1.3%
AMX0010C	GHA	77.2%	±	0.6%	0.1%	1	0	3%	254	±	2	1.1%	252	8	3%	101.8%	±	0.8%
AMX0011C	GHA	75.3%	±	1.8%	-1.5%	1	0	1%	245	±	6	0.3%	244	1	1%	97.9%	±	2.3%
AMX0011C	GHA	76.5%	±	0.2%	0.0%	1	0	1%	243	±	0	-0.5%	244	1	1%	97.0%	±	0.2%
AMX0011C	GHA	77.7%	±	0.2%	1.6%	1	0	1%	246	±	1	0.8%	244	1	1%	98.3%	±	0.2%
AMX0011C	GHA	76.9%	±	0.6%	0.6%	1	0	1%	243	±	2	-0.2%	244	1	1%	97.3%	±	0.7%
AMX0011C	GHA	75.9%	±	0.2%	-0.8%	1	0	1%	243	±	1	-0.3%	244	1	1%	97.3%	±	0.2%
AMX0012	DRC	86.6%	±	0.6%	-0.5%	1	0	3%	448	±	3	-4.6%	469	30	6%	89.5%	±	0.6%
AMX0012	DRC	84%	±	1%	-3.1%	1	0	3%	464	±	6	-1.0%	469	30	6%	92.9%	±	1.1%
AMX0012	DRC	86%	±	4%	-0.8%	1	0	3%	434	±	18	-7.5%	469	30	6%	86.8%	±	3.6%

UPLC-UV analysis

AMX0012	DRC	88%	±	1%	0.5%	1	0	3%	502	±	8	7.0%	469	30	6%	100.4%	±	1.6%
AMX0012	DRC	90.4%	±	0.7%	3.9%	1	0	3%	497	±	4	6.0%	469	30	6%	99.5%	±	0.7%
AMX0023A	HAI	76%	±	1%	2.8%	1	0	7%	471	±	8	19.0%	396	59	15%	94.1%	±	1.7%
AMX0023A	HAI	77%	±	2%	3.3%	1	0	7%	443	±	11	12.1%	396	59	15%	88.7%	±	2.3%
AMX0023A	HAI	80.3%	±	3.2%	8.2%	1	0	7%	375	±	15	-5.2%	396	59	15%	75.0%	±	2.9%
AMX0023A	HAI	71%	±	2%	-4.0%	1	0	7%	334	±	8	-15.5%	396	59	15%	66.9%	±	1.7%
AMX0023A	HAI	67%	±	4%	-10.3%	1	0	7%	355	±	23	-10.4%	396	59	15%	70.9%	±	4.6%
AMX0023B	HAI	81%	±	0.9%	-2.3%	1	0	3%	490	±	6	-2.5%	503	18	4%	98.0%	±	1.1%
AMX0023B	HAI	82%	±	1.1%	-1.1%	1	0	3%	484	±	7	-3.7%	503	18	4%	96.7%	±	1.3%
AMX0023B	HAI	85%	±	0.6%	2.8%	1	0	3%	530	±	4	5.4%	503	18	4%	105.9%	±	0.7%
AMX0023B	HAI	85%	±	0.6%	2.8%	1	0	3%	502	±	3	-0.1%	503	18	4%	100.5%	±	0.7%
AMX0023B	HAI	81%	±	0.5%	-2.3%	1	0	3%	507	±	3	0.9%	503	18	4%	101.4%	±	0.6%
AMX0024A	HAI	81%	±	1%	-0.8%	1	0	5%	494	±	4	0.7%	491	31	6%	98.8%	±	0.9%
AMX0024A	HAI	77%	±	2%	-5.5%	1	0	5%	472	±	15	-3.8%	491	31	6%	94.4%	±	3.0%
AMX0024A	HAI	81.2%	±	0.6%	-0.6%	1	0	5%	448	±	3	-8.7%	491	31	6%	89.6%	±	0.7%
AMX0024A	HAI	81%	±	1%	-0.7%	1	0	5%	513	±	4	4.5%	491	31	6%	102.5%	±	0.9%
AMX0024A	HAI	88%	±	1%	7.7%	1	0	5%	526	±	6	7.3%	491	31	6%	105.3%	±	1.2%
AMX0024B	HAI	78%	±	5%	-0.5%	1	0	3%	467	±	30	8.2%	431	68	16%	93.3%	±	6.0%
AMX0024B	HAI	81%	±	2%	3.3%	1	0	3%	310	±	8	-28.0%	431	68	16%	62.1%	±	1.5%
AMX0024B	HAI	81.0%	±	0%	3.3%	1	0	3%	469	±	0	8.8%	431	68	16%	93.8%	±	0.0%
AMX0024B	HAI	76%	±	3%	-3.1%	1	0	3%	461	±	18	6.8%	431	68	16%	92.2%	±	3.6%
AMX0024B	HAI	76%	±	2%	-3.1%	1	0	3%	449	±	12	4.2%	431	68	16%	89.9%	±	2.4%
AMX0025	IND	81%	±	0.00%	0.2%	1	0	1%	487	±	0	-0.2%	487	5	1%	97.3%	±	0.0%
AMX0025	IND	80%	±	2.0%	-1.3%	1	0	1%	480	±	12	-1.5%	487	5	1%	96.0%	±	2.4%
AMX0025	IND	81%	±	1.0%	-0.1%	1	0	1%	489	±	6	0.3%	487	5	1%	97.7%	±	1.2%
AMX0025	IND	81%	±	1%	-0.1%	1	0	1%	494	±	6	1.3%	487	5	1%	98.7%	±	1.2%
AMX0025	IND	82%	±	0.0%	1.2%	1	0	1%	488	±	0	0.1%	487	5	1%	97.6%	±	0.0%
AMX0027	PNG	75.0%	±	0.8%	-1.3%	1	0	3%	472	±	5	-0.6%	475	13	3%	94.4%	±	1.0%
AMX0027	PNG	73%	±	5%	-3.9%	1	0	3%	462	±	34	-2.7%	475	13	3%	92.4%	±	6.8%
AMX0027	PNG	77.0%	±	1.1%	1.3%	1	0	3%	483	±	7	1.7%	475	13	3%	96.6%	±	1.4%
AMX0027	PNG	79%	±	1%	3.9%	1	0	3%	492	±	6	3.7%	475	13	3%	98.5%	±	1.1%
AMX0027	PNG	76.0%	±	1.0%	0.0%	1	0	3%	464	±	6	-2.2%	475	13	3%	92.8%	±	1.2%
AMX0028	ETH	84.0%	±	4.1%	-2.1%	1	0	2%	503	±	25	-0.5%	506	16	3%	100.6%	±	4.9%
AMX0028	ETH	86%	±	0%	0.2%	1	0	2%	525	±	0	3.7%	506	16	3%	105.0%	±	0.1%

UPLC-UV analysis

AMX0028	ETH	85.0%	±	1.3%	-0.9%	1	0	2%	481	±	7	-5.0%	506	16	3%	96.1%	±	1.4%
AMX0028	ETH	86%	±	2%	0.2%	1	0	2%	508	±	13	0.5%	506	16	3%	101.7%	±	2.6%
AMX0028	ETH	88.0%	±	0.3%	2.6%	1	0	2%	513	±	2	1.4%	506	16	3%	102.6%	±	0.3%
AMX0029A	ETH	88.0%	±	0.4%	1.4%	1	0	2%	505	±	2	1.7%	497	8	2%	101.1%	±	0.5%
AMX0029A	ETH	87.0%	±	0.5%	0.2%	1	0	2%	500	±	3	0.5%	497	8	2%	99.9%	±	0.6%
AMX0029A	ETH	88.00%	±	1.43%	1.4%	1	0	2%	502	±	8	1.0%	497	8	2%	100.4%	±	1.6%
AMX0029A	ETH	86.0%	±	1.3%	-0.9%	1	0	2%	492	±	7	-0.9%	497	8	2%	98.4%	±	1.4%
AMX0029A	ETH	85.00%	±	0.02%	-2.1%	1	0	2%	485	±	0	-2.4%	497	8	2%	97.0%	±	0.0%
AMX0029B	ETH	86.4%	±	0.3%	-0.2%	1	0	1%	495	±	2	-0.7%	498	6	1%	99.0%	±	0.3%
AMX0029B	ETH	86%	±	0%	-0.3%	1	0	1%	497	±	2	-0.3%	498	6	1%	99.3%	±	0.3%
AMX0029B	ETH	87.8%	±	0.2%	1.4%	1	0	1%	508	±	1	1.9%	498	6	1%	101.6%	±	0.2%
AMX0029B	ETH	86%	±	3%	-1.1%	1	0	1%	494	±	15	-1.0%	498	6	1%	98.7%	±	3.1%
AMX0029B	ETH	87%	±	1%	0.2%	1	0	1%	499	±	3	0.1%	498	6	1%	99.8%	±	0.6%
AMX0031A	SLE	78.3%	±	0.1%	0.3%	1	0	1%	239	±	0	-2.3%	244	5	2%	95.5%	±	0.1%
AMX0031A	SLE	76.5%	±	0.1%	-2.1%	1	0	1%	239	±	0	-2.1%	244	5	2%	95.7%	±	0.1%
AMX0031A	SLE	78.7%	±	0.1%	0.8%	1	0	1%	246	±	0	0.5%	244	5	2%	98.3%	±	0.1%
AMX0031A	SLE	79.0%	±	0.1%	1.1%	1	0	1%	251	±	0	2.7%	244	5	2%	100.5%	±	0.1%
AMX0031A	SLE	78.0%	±	0.0%	-0.1%	1	0	1%	247	±	0	1.2%	244	5	2%	99.0%	±	0.1%
AMX0031B	SLE	79.6%	±	0.2%	2.3%	1	0	3%	254	±	1	3.8%	245	10	4%	101.6%	±	0.3%
AMX0031B	SLE	79.3%	±	0.2%	1.9%	1	0	3%	256	±	1	4.6%	245	10	4%	102.4%	±	0.3%
AMX0031B	SLE	79.1%	±	0.0%	1.6%	1	0	3%	242	±	0	-0.9%	245	10	4%	97.0%	±	0.0%
AMX0031B	SLE	75.8%	±	0.0%	-2.6%	1	0	3%	238	±	0	-2.8%	245	10	4%	95.1%	±	0.0%
AMX0031B	SLE	75.3%	±	0.1%	-3.2%	1	0	3%	233	±	0	-4.7%	245	10	4%	93.2%	±	0.1%
AMX0031C	SLE	74.8%	±	0.1%	-2.4%	1	0	3%	236	±	0	-0.4%	237	8	3%	94.5%	±	0.1%
AMX0031C	SLE	75.9%	±	0.1%	-1.0%	1	0	3%	236	±	0	-0.6%	237	8	3%	94.3%	±	0.1%
AMX0031C	SLE	73.8%	±	0.2%	-3.7%	1	0	3%	226	±	1	-4.8%	237	8	3%	90.4%	±	0.2%
AMX0031C	SLE	79.2%	±	0.3%	3.3%	1	0	3%	240	±	1	1.1%	237	8	3%	96.0%	±	0.4%
AMX0031C	SLE	79.5%	±	0.3%	3.7%	1	0	3%	249	±	1	4.8%	237	8	3%	99.5%	±	0.4%
AMX0032	SLE	85.1%	±	0.1%	-0.3%	1	0	2%	500	±	1	-0.4%	502	13	3%	100.0%	±	0.1%
AMX0032	SLE	85.0%	±	0.0%	-0.4%	1	0	2%	490	±	0	-2.3%	502	13	3%	98.1%	±	0.0%
AMX0032	SLE	86.3%	±	0.0%	1.1%	1	0	2%	510	±	0	1.7%	502	13	3%	102.1%	±	0.0%
AMX0032	SLE	86.9%	±	0.0%	1.8%	1	0	2%	519	±	0	3.4%	502	13	3%	103.8%	±	0.0%
AMX0032	SLE	83.4%	±	0.0%	-2.3%	1	0	2%	490	±	0	-2.4%	502	13	3%	97.9%	±	0.0%
AMX0033A	SLE	84.7%	±	0.0%	-0.6%	1	0	1%	507	±	0	-0.8%	511	5	1%	101.4%	±	0.0%

UPLC-UV analysis

AMX0033A	SLE	84.7%	±	0.0%	-0.6%	1	0	1%	510	±	0	-0.2%	511	5	1%	102.0%	±	0.0%
AMX0033A	SLE	85.5%	±	0.0%	0.3%	1	0	1%	508	±	0	-0.7%	511	5	1%	101.5%	±	0.0%
AMX0033A	SLE	84.7%	±	0.0%	-0.6%	1	0	1%	513	±	0	0.3%	511	5	1%	102.5%	±	0.0%
AMX0033A	SLE	86.5%	±	0.0%	1.5%	1	0	1%	518	±	0	1.4%	511	5	1%	103.6%	±	0.0%
AMX0033B	SLE	84.7%	±	0.1%	-0.3%	1	0	2%	509	±	1	-1.3%	516	16	3%	101.9%	±	0.1%
AMX0033B	SLE	84.5%	±	0.0%	-0.5%	1	0	2%	510	±	0	-1.1%	516	16	3%	102.0%	±	0.0%
AMX0033B	SLE	85.5%	±	0.1%	0.6%	1	0	2%	526	±	1	2.0%	516	16	3%	105.2%	±	0.1%
AMX0033B	SLE	83.1%	±	0.0%	-2.2%	1	0	2%	496	±	0	-3.8%	516	16	3%	99.2%	±	0.0%
AMX0033B	SLE	87.0%	±	0.0%	2.4%	1	0	2%	537	±	0	4.2%	516	16	3%	107.5%	±	0.0%
AMX0033C	SLE	86.5%	±	0.0%	-0.5%	1	0	2%	514	±	0	-1.6%	523	11	2%	102.8%	±	0.0%
AMX0033C	SLE	87.5%	±	0.0%	0.7%	1	0	2%	517	±	0	-1.1%	523	11	2%	103.4%	±	0.0%
AMX0033C	SLE	87.8%	±	0.0%	1.0%	1	0	2%	533	±	0	2.1%	523	11	2%	106.7%	±	0.0%
AMX0033C	SLE	84.3%	±	0.0%	-3.0%	1	0	2%	512	±	0	-1.9%	523	11	2%	102.5%	±	0.0%
AMX0033C	SLE	88.4%	±	0.0%	1.7%	1	0	2%	536	±	0	2.5%	523	11	2%	107.1%	±	0.0%
AMX0033D	SLE	84.9%	±	0.0%	1.5%	1	0	2%	519	±	0	2.4%	507	10	2%	103.9%	±	0.0%
AMX0033D	SLE	86.3%	±	0.1%	3.1%	1	0	2%	516	±	1	1.7%	507	10	2%	103.1%	±	0.1%
AMX0033D	SLE	82.4%	±	0.2%	-1.5%	1	0	2%	500	±	1	-1.4%	507	10	2%	100.0%	±	0.2%
AMX0033D	SLE	81.8%	±	0.0%	-2.2%	1	0	2%	499	±	0	-1.6%	507	10	2%	99.8%	±	0.0%
AMX0033D	SLE	83.0%	±	0.0%	-0.8%	1	0	2%	501	±	0	-1.1%	507	10	2%	100.3%	±	0.0%
AMX0034A	SLE	78.6%	±	0.2%	-3.2%	1	0	2%	470	±	1	-4.1%	490	13	3%	93.9%	±	0.2%
AMX0034A	SLE	81.0%	±	0.0%	-0.3%	1	0	2%	488	±	0	-0.3%	490	13	3%	97.6%	±	0.0%
AMX0034A	SLE	81.4%	±	0.0%	0.2%	1	0	2%	489	±	0	0.0%	490	13	3%	97.9%	±	0.0%
AMX0034A	SLE	83.0%	±	0.0%	2.2%	1	0	2%	507	±	0	3.5%	490	13	3%	101.4%	±	0.0%
AMX0034A	SLE	82.1%	±		1.1%	1	0	2%	494	±	0	0.9%	490	13	3%	98.8%	±	0.0%
AMX0034B	SLE	81.4%	±	0.0%	-0.1%	1	0	5%	488	±	0	0.2%	487	24	5%	97.6%	±	0.0%
AMX0034B	SLE	84.1%	±	0.0%	3.2%	1	0	5%	505	±	0	3.6%	487	24	5%	100.9%	±	0.0%
AMX0034B	SLE	74.9%	±	0.1%	-8.1%	1	0	5%	449	±	1	-7.8%	487	24	5%	89.8%	±	0.1%
AMX0034B	SLE	81.9%	±	0.1%	0.5%	1	0	5%	482	±	1	-1.0%	487	24	5%	96.4%	±	0.1%
AMX0034B	SLE	85.0%	±	0.0%	4.3%	1	0	5%	511	±	0	5.0%	487	24	5%	102.3%	±	0.0%
AMX0035A	SLE	82.9%	±	0.3%	-3.3%	1	0	5%	477	±	2	-4.5%	500	27	5%	95.4%	±	0.3%
AMX0035A	SLE	89.4%	±	0.3%	4.2%	1	0	5%	519	±	2	3.9%	500	27	5%	103.8%	±	0.3%
AMX0035A	SLE	88.5%	±	0.2%	3.2%	1	0	5%	500	±	1	0.1%	500	27	5%	100.0%	±	0.2%
AMX0035A	SLE	89.1%	±	0.3%	3.9%	1	0	5%	534	±	2	6.8%	500	27	5%	106.7%	±	0.4%
AMX0035A	SLE	78.9%	±	0.0%	-8.0%	1	0	5%	469	±	0	-6.2%	500	27	5%	93.7%	±	0.0%

UPLC-UV analysis

AMX0035B	SLE	88.6%	±	0.0%	4.6%	1	0	3%	504	±	0	3.2%	489	10	2%	100.9%	±	0.0%
AMX0035B	SLE	83.3%	±	0.0%	-1.6%	1	0	3%	478	±	0	-2.1%	489	10	2%	95.7%	±	0.0%
AMX0035B	SLE	81.7%	±	0.1%	-3.5%	1	0	3%	488	±	1	-0.2%	489	10	2%	97.5%	±	0.1%
AMX0035B	SLE	84.7%	±	0.1%	0.0%	1	0	3%	488	±	1	-0.1%	489	10	2%	97.7%	±	0.1%
AMX0035B	SLE	85.1%	±	0.2%	0.5%	1	0	3%	484	±	1	-0.9%	489	10	2%	96.9%	±	0.2%
AMX0036A	SLE	83.5%	±	0.2%	1.6%	1	0	2%	248	±	1	4.9%	237	9	4%	99.2%	±	0.2%
AMX0036A	SLE	83.4%	±	0.0%	1.5%	1	0	2%	241	±	0	2.0%	237	9	4%	96.5%	±	0.0%
AMX0036A	SLE	81.8%	±	0.2%	-0.5%	1	0	2%	237	±	1	0.4%	237	9	4%	95.0%	±	0.2%
AMX0036A	SLE	82.1%	±	0.1%	-0.1%	1	0	2%	229	±	0	-3.2%	237	9	4%	91.6%	±	0.1%
AMX0036A	SLE	80.1%	±	0.0%	-2.5%	1	0	2%	227	±	0	-4.1%	237	9	4%	90.7%	±	0.0%
AMX0036B	SLE	78.0%	±	0.2%	-2.0%	1	0	2%	196	±	1	-15.3%	231	22	9%	78.2%	±	0.2%
AMX0036B	SLE	79.3%	±	0.0%	-0.4%	1	0	2%	230	±	0	-0.2%	231	22	9%	92.2%	±	0.0%
AMX0036B	SLE	78.9%	±	0.2%	-0.9%	1	0	2%	244	±	1	5.5%	231	22	9%	97.4%	±	0.2%
AMX0036B	SLE	81.1%	±	0.1%	1.9%	1	0	2%	232	±	0	0.6%	231	22	9%	92.9%	±	0.1%
AMX0036B	SLE	80.6%	±	0.1%	1.3%	1	0	2%	252	±	0	9.3%	231	22	9%	101.0%	±	0.1%
AMX0037A	SLE	84.4%	±	0.0%	1.1%	1	0	2%	489	±	0	0.1%	489	16	3%	97.9%	±	0.0%
AMX0037A	SLE	84.8%	±	0.2%	1.6%	1	0	2%	465	±	1	-4.9%	489	16	3%	93.0%	±	0.2%
AMX0037A	SLE	80.9%	±	0.0%	-3.1%	1	0	2%	492	±	0	0.7%	489	16	3%	98.4%	±	0.0%
AMX0037A	SLE	82.8%	±	0.0%	-0.8%	1	0	2%	488	±	0	-0.2%	489	16	3%	97.6%	±	0.0%
AMX0037A	SLE	84.4%	±	0.0%	1.1%	1	0	2%	509	±	0	4.2%	489	16	3%	101.9%	±	0.0%
AMX0038A	SLE	80.1%	±	0.0%	1.4%	1	0	2%	231	±	0	1.1%	228	3	1%	92.4%	±	0.0%
AMX0038A	SLE	77.3%	±	0.0%	-2.1%	1	0	2%	227	±	0	-0.8%	228	3	1%	90.7%	±	0.0%
AMX0038A	SLE	78.0%	±	0.0%	-1.2%	1	0	2%	225	±	0	-1.4%	228	3	1%	90.1%	±	0.0%
AMX0038A	SLE	79.5%	±	0.0%	0.7%	1	0	2%	228	±	0	-0.3%	228	3	1%	91.1%	±	0.0%
AMX0038A	SLE	79.9%	±	0.0%	1.2%	1	0	2%	232	±	0	1.4%	228	3	1%	92.7%	±	0.0%
AMX0038B	SLE	77.0%	±	0.3%	-3.6%	1	0	2%	222	±	1	-3.1%	229	4	2%	88.7%	±	0.3%
AMX0038B	SLE	81.0%	±	0.0%	1.4%	1	0	2%	234	±	0	2.1%	229	4	2%	93.5%	±	0.0%
AMX0038B	SLE	80.3%	±	0.2%	0.5%	1	0	2%	230	±	1	0.4%	229	4	2%	91.9%	±	0.2%
AMX0038B	SLE	80.2%	±	0.2%	0.4%	1	0	2%	230	±	1	0.4%	229	4	2%	91.9%	±	0.2%
AMX0038B	SLE	80.9%	±	0.0%	1.3%	1	0	2%	229	±	0	0.2%	229	4	2%	91.7%	±	0.0%
AMX0039A	SLE	81.8%	±	0.0%	1.1%	1	0	1%	504	±	0	3.3%	488	11	2%	100.8%	±	0.0%
AMX0039A	SLE	81.5%	±	0.0%	0.7%	1	0	1%	486	±	0	-0.4%	488	11	2%	97.2%	±	0.0%
AMX0039A	SLE	81.3%	±	0.0%	0.5%	1	0	1%	485	±	0	-0.6%	488	11	2%	97.0%	±	0.0%
AMX0039A	SLE	79.2%	±	0.0%	-2.1%	1	0	1%	474	±	0	-2.9%	488	11	2%	94.8%	±	0.0%

UPLC-UV analysis

AMX0039A	SLE	80.8%	±	0.0%	-0.1%	1	0	1%	490	±	0	0.5%	488	11	2%	98.1%	±	0.0%
AMX0039B	SLE	80.9%	±	0.0%	-1.8%	1	0	1%	495	±	0	-1.4%	502	9	2%	98.9%	±	0.0%
AMX0039B	SLE	82.4%	±	0.0%	0.0%	1	0	1%	496	±	0	-1.1%	502	9	2%	99.2%	±	0.0%
AMX0039B	SLE	83.1%	±	0.0%	0.8%	1	0	1%	495	±	0	-1.3%	502	9	2%	99.1%	±	0.0%
AMX0039B	SLE	82.9%	±	0.0%	0.6%	1	0	1%	514	±	0	2.5%	502	9	2%	102.9%	±	0.0%
AMX0039B	SLE	82.7%	±	0.0%	0.4%	1	0	1%	508	±	0	1.3%	502	9	2%	101.6%	±	0.0%
AMX0040A	SLE	82.6%	±	0.2%	4.6%	1	0	3%	494	±	1	4.6%	472	17	4%	98.9%	±	0.2%
AMX0040A	SLE	79.9%	±	0.0%	1.2%	1	0	3%	472	±	0	-0.1%	472	17	4%	94.4%	±	0.0%
AMX0040A	SLE	75.0%	±	0.0%	-5.0%	1	0	3%	446	±	0	-5.5%	472	17	4%	89.3%	±	0.0%
AMX0040A	SLE	78.7%	±	0.0%	-0.3%	1	0	3%	473	±	0	0.0%	472	17	4%	94.5%	±	0.0%
AMX0040A	SLE	78.5%	±	0.1%	-0.6%	1	0	3%	477	±	1	1.0%	472	17	4%	95.4%	±	0.1%
AMX0040B	SLE	83.1%	±	0.2%	0.9%	1	0	1%	481	±	1	-1.2%	487	7	1%	96.2%	±	0.2%
AMX0040B	SLE	81.9%	±	0.2%	-0.5%	1	0	1%	484	±	1	-0.5%	487	7	1%	96.9%	±	0.2%
AMX0040B	SLE	82.6%	±	0.0%	0.3%	1	0	1%	494	±	0	1.4%	487	7	1%	98.7%	±	0.0%
AMX0040B	SLE	81.7%	±	0.2%	-0.8%	1	0	1%	480	±	1	-1.3%	487	7	1%	96.1%	±	0.2%
AMX0040B	SLE	82.3%	±	0.2%	0.0%	1	0	1%	494	±	1	1.5%	487	7	1%	98.8%	±	0.2%
AMX0041	GHA	81.5%	±	0.1%	-0.8%	1	0	3%	490	±	1	0.2%	489	16	3%	98.0%	±	0.1%
AMX0041	GHA	80.0%	±	0.1%	-2.6%	1	0	3%	474	±	1	-3.1%	489	16	3%	94.8%	±	0.1%
AMX0041	GHA	81.0%	±	0.5%	-1.4%	1	0	3%	477	±	3	-2.4%	489	16	3%	95.4%	±	0.6%
AMX0041	GHA	82.6%	±	0.3%	0.6%	1	0	3%	488	±	2	-0.2%	489	16	3%	97.6%	±	0.4%
AMX0041	GHA	85.5%	±	0.0%	4.1%	1	0	3%	516	±	0	5.4%	489	16	3%	103.1%	±	0.0%
AMX0042	GHA	86.5%	±	0.0%	1.1%	1	0	1%	508	±	0	0.3%	506	7	1%	101.5%	±	0.0%
AMX0042	GHA	86.7%	±	0.0%	1.4%	1	0	1%	516	±	0	2.0%	506	7	1%	103.2%	±	0.0%
AMX0042	GHA	85.9%	±	0.0%	0.4%	1	0	1%	509	±	0	0.5%	506	7	1%	101.7%	±	0.0%
AMX0042	GHA	84.8%	±	0.1%	-0.9%	1	0	1%	502	±	1	-0.8%	506	7	1%	100.5%	±	0.1%
AMX0042	GHA	83.8%	±	0.0%	-2.0%	1	0	1%	496	±	0	-2.0%	506	7	1%	99.2%	±	0.0%
AMX0043	GHA	84.4%	±	0.0%	-1.7%	1	0	1%	494	±	0	-2.7%	508	9	2%	98.8%	±	0.0%
AMX0043	GHA	84.9%	±	0.0%	-1.1%	1	0	1%	511	±	0	0.5%	508	9	2%	102.1%	±	0.0%
AMX0043	GHA	87.7%	±	0.0%	2.2%	1	0	1%	519	±	0	2.2%	508	9	2%	103.8%	±	0.0%
AMX0043	GHA	85.9%	±	0.1%	0.1%	1	0	1%	508	±	1	0.1%	508	9	2%	101.6%	±	0.1%
AMX0043	GHA	86.2%	±	0.0%	0.4%	1	0	1%	508	±	0	-0.1%	508	9	2%	101.5%	±	0.0%
AMX0044	GHA	86.4%	±	0.0%	4.2%	1	0	4%	521	±	0	4.9%	496	21	4%	104.1%	±	0.0%
AMX0044	GHA	85.4%	±	0.0%	3.0%	1	0	4%	511	±	0	3.0%	496	21	4%	102.2%	±	0.0%
AMX0044	GHA	78.3%	±	0.0%	-5.5%	1	0	4%	465	±	0	-6.2%	496	21	4%	93.1%	±	0.0%

UPLC-UV analysis

AMX0044	GHA	83.3%	±	0.0%	0.5%	1	0	4%	497	±	0	0.2%	496	21	4%	99.4%	±	0.0%
AMX0044	GHA	81.0%	±	0.0%	-2.3%	1	0	4%	487	±	0	-1.8%	496	21	4%	97.4%	±	0.0%
AMX0045	GHA	86.2%	±	0.1%	1.9%	1	0	2%	500	±	1	5.3%	475	20	4%	100.1%	±	0.1%
AMX0045	GHA	85.5%	±	0.1%	1.1%	1	0	2%	479	±	1	0.7%	475	20	4%	95.8%	±	0.1%
AMX0045	GHA	84.2%	±	0.0%	-0.4%	1	0	2%	482	±	0	1.4%	475	20	4%	96.4%	±	0.0%
AMX0045	GHA	85.2%	±	0.0%	0.8%	1	0	2%	468	±	0	-1.6%	475	20	4%	93.5%	±	0.0%
AMX0045	GHA	81.7%	±	0.0%	-3.4%	1	0	2%	448	±	0	-5.8%	475	20	4%	89.5%	±	0.0%
AMX0046	GHA	79.0%	±	0.0%	3.6%	1	0	4%	491	±	0	2.4%	479	13	3%	98.1%	±	0.0%
AMX0046	GHA	80.3%	±	0.0%	5.3%	1	0	4%	493	±	0	2.9%	479	13	3%	98.7%	±	0.0%
AMX0046	GHA	72.6%	±	0.0%	-4.8%	1	0	4%	464	±	0	-3.2%	479	13	3%	92.8%	±	0.0%
AMX0046	GHA	74.9%	±	0.2%	-1.8%	1	0	4%	467	±	1	-2.6%	479	13	3%	93.3%	±	0.2%
AMX0046	GHA	74.5%	±	0.0%	-2.3%	1	0	4%	481	±	0	0.4%	479	13	3%	96.3%	±	0.0%
AMX0047	GHA	84.2%	±	0.1%	0.0%	1	0	1%	464	±	1	-5.8%	493	17	3%	92.9%	±	0.1%
AMX0047	GHA	85.2%	±	0.1%	1.2%	1	0	1%	500	±	1	1.4%	493	17	3%	100.0%	±	0.1%
AMX0047	GHA	85.0%	±	0.1%	1.0%	1	0	1%	510	±	1	3.5%	493	17	3%	102.0%	±	0.1%
AMX0047	GHA	82.9%	±	0.0%	-1.5%	1	0	1%	494	±	0	0.3%	493	17	3%	98.9%	±	0.0%
AMX0047	GHA	83.6%	±	0.0%	-0.7%	1	0	1%	497	±	0	0.7%	493	17	3%	99.3%	±	0.0%
AMX0048A	HAI	83.1%	±	0.1%	1.6%	1	0	2%	483	±	1	-0.4%	485	13	3%	96.6%	±	0.1%
AMX0048A	HAI	80.2%	±	0.0%	-2.0%	1	0	2%	465	±	0	-4.1%	485	13	3%	92.9%	±	0.0%
AMX0048A	HAI	79.8%	±	0.1%	-2.4%	1	0	2%	484	±	1	-0.2%	485	13	3%	96.8%	±	0.1%
AMX0048A	HAI	82.0%	±	0.0%	0.2%	1	0	2%	495	±	0	2.0%	485	13	3%	98.9%	±	0.0%
AMX0048A	HAI	83.9%	±	0.0%	2.6%	1	0	2%	498	±	0	2.7%	485	13	3%	99.5%	±	0.0%
AMX0048B	HAI	83.0%	±	0.1%	0.8%	1	0	1%	487	±	1	1.4%	480	18	4%	97.3%	±	0.1%
AMX0048B	HAI	83.0%	±	0.1%	0.8%	1	0	1%	500	±	1	4.4%	480	18	4%	100.1%	±	0.1%
AMX0048B	HAI	80.9%	±	0.0%	-1.8%	1	0	1%	454	±	0	-5.3%	480	18	4%	90.9%	±	0.0%
AMX0048B	HAI	81.6%	±	0.0%	-0.9%	1	0	1%	470	±	0	-2.1%	480	18	4%	94.0%	±	0.0%
AMX0048B	HAI	83.4%	±	0.0%	1.2%	1	0	1%	487	±	0	1.5%	480	18	4%	97.4%	±	0.0%
AMX0048C	HAI	82.8%	±	0.0%	-1.3%	1	0	1%	484	±	0	-2.1%	495	12	2%	96.8%	±	0.0%
AMX0048C	HAI	83.5%	±	0.2%	-0.5%	1	0	1%	489	±	1	-1.1%	495	12	2%	97.9%	±	0.2%
AMX0048C	HAI	85.7%	±	0.2%	2.1%	1	0	1%	513	±	1	3.7%	495	12	2%	102.6%	±	0.2%
AMX0048C	HAI	83.4%	±	0.0%	-0.6%	1	0	1%	488	±	0	-1.4%	495	12	2%	97.6%	±	0.0%
AMX0048C	HAI	84.2%	±	0.1%	0.3%	1	0	1%	499	±	1	0.8%	495	12	2%	99.8%	±	0.1%
AMX0050A	DRC	46%	±	2%	0.0%	0	0	2%	245	±	10	-0.8%	247	14	5%	97.9%	±	3.8%
AMX0050A	DRC	45%	±	5%	-2.3%	0	0	2%	225	±	26	-8.8%	247	14	5%	90.0%	±	10.4%

UPLC-UV analysis

AMX0050A	DRC	46%	±	2%	0.4%	0	0	2%	256	±	10	3.7%	247	14	5%	102.3%	±	4.1%
AMX0050A	DRC	47%	±	2%	2.0%	0	0	2%	260	±	9	5.3%	247	14	5%	103.9%	±	3.4%
AMX0050A	DRC	45.98%	±	0.03%	-0.1%	0	0	2%	249	±	0	0.7%	247	14	5%	99.4%	±	0.1%
AMX0050B	DRC	47%	±	6%	5.6%	0	0	5%	238	±	28	0.3%	237	9	4%	95.2%	±	11.1%
AMX0050B	DRC	44%	±	1%	-2.3%	0	0	5%	227	±	5	-4.3%	237	9	4%	90.8%	±	2.0%
AMX0050B	DRC	43.8%	±	0.1%	-2.0%	0	0	5%	245	±	0	3.3%	237	9	4%	98.1%	±	0.1%
AMX0050B	DRC	42%	±	1%	-6.0%	0	0	5%	230	±	5	-3.1%	237	9	4%	92.1%	±	2.2%
AMX0050B	DRC	46.8%	±	0.9%	4.8%	0	0	5%	247	±	5	3.8%	237	9	4%	98.6%	±	1.8%
AMX0051A	DRC	79%	±	2%	1.1%	1	0	2%	478	±	15	1.4%	471	12	3%	95.6%	±	3.0%
AMX0051A	DRC	80%	±	1%	2.9%	1	0	2%	485	±	4	2.9%	471	12	3%	97.0%	±	0.8%
AMX0051A	DRC	79%	±		1.1%	1	0	2%	476	±	0	1.1%	471	12	3%	95.3%	±	0.0%
AMX0051A	DRC	76%	±	4%	-2.8%	1	0	2%	456	±	27	-3.2%	471	12	3%	91.3%	±	5.3%
AMX0051A	DRC	76%	±	2%	-2.4%	1	0	2%	461	±	14	-2.2%	471	12	3%	92.1%	±	2.9%
AMX0051B	DRC	78.8%	±	0.3%	2.2%	1	0	2%	475	±	2	2.3%	465	8	2%	95.0%	±	0.3%
AMX0051B	DRC	75%	±	1%	-2.1%	1	0	2%	455	±	6	-2.1%	465	8	2%	90.9%	±	1.3%
AMX0051B	DRC	76.5%	±	0.3%	-0.7%	1	0	2%	464	±	2	-0.2%	465	8	2%	92.7%	±	0.4%
AMX0051B	DRC	78%	±	2%	0.6%	1	0	2%	465	±	9	0.1%	465	8	2%	93.0%	±	1.8%

FT-IR Analyses

Sample #	SAMP. LOC.	Measured %w/w			%w/w			RMD	AVERAGE	STDEV	STDEV%	API mass			RMD	AVERAGE	STDEV	STDEV%	%API		
AMXSTD1	CANADA	85.9242	84.6922	82.2668	84.3%	±	1.9%	0.3%	1	0	3%	525	±	5	0.5%	522	20	4%	104.9%	±	0.9%
AMXSTD1	CANADA	80.8520	80.5096	82.1170	81.2%	±	0.8%	-3.5%	1	0	3%	505	±	6	-3.3%	522	20	4%	100.9%	±	1.1%
AMXSTD1	CANADA	83.9144	81.3553	81.5792	82.3%	±	1.4%	-2.1%	1	0	3%	520	±	7	-0.3%	522	20	4%	104.0%	±	1.3%
AMXSTD1	CANADA	83.2528	82.3550	83.0088	82.9%	±	0.5%	-1.4%	1	0	3%	508	±	8	-2.6%	522	20	4%	101.7%	±	1.5%
AMXSTD1	CANADA	81.6699	84.7056	82.4152	82.9%	±	1.6%	-1.3%	1	0	3%	513	±	9	-1.7%	522	20	4%	102.6%	±	1.7%
AMXSTD1	CANADA	82.5571	81.9539	82.1554	82.2%	±	0.3%	-2.2%	1	0	3%	497	±	10	-4.8%	522	20	4%	99.4%	±	1.9%
AMXSTD1	CANADA	83.9883	82.973	86.6913	84.6%	±	1.9%	0.6%	1	0	3%	525	±	11	0.5%	522	20	4%	104.9%	±	2.1%
AMXSTD1	CANADA	91.5734	87.3105	86.9623	88.6%	±	2.6%	5.4%	1	0	3%	557	±	12	6.8%	522	20	4%	111.5%	±	2.3%
AMXSTD1	CANADA	85.6886	86.2307	90.9365	87.6%	±	2.9%	4.2%	1	0	3%	547	±	13	4.8%	522	20	4%	109.4%	±	2.5%
AMX0001	DRC	90.8273	88.5894	85.0011	88.1%	±	2.9%	-0.7%	1	0	1%	497	±	17	1.3%	491	15	3%	99.4%	±	3.3%
AMX0001	DRC	89.4297	89.6245	85.4948	88.2%	±	2.3%	-0.7%	1	0	1%	501	±	13	2.0%	491	15	3%	100.1%	±	2.6%
AMX0001	DRC	88.6222	87.6105	92.935	89.7%	±	2.8%	1.1%	1	0	1%	481	±	15	-2.0%	491	15	3%	96.2%	±	3.0%
AMX0001	DRC	87.5334	86.6278	90.8481	88.3%	±	2.2%	-0.5%	1	0	1%	470	±	12	-4.3%	491	15	3%	93.9%	±	2.4%
AMX0001	DRC	89.9849	86.4407	92.1773	89.5%	±	2.9%	0.8%	1	0	1%	505	±	16	3.0%	491	15	3%	101.1%	±	3.3%
AMX0002	DRC	84.6103	81.9388	83.9747	83.5%	±	1.4%	-2.5%	1	0	3%	467	±	8	-3.4%	483	14	3%	93.4%	±	1.6%
AMX0002	DRC	85.0907	87.9553	94.8715	89.3%	±	5.0%	4.2%	1	0	3%	495	±	28	2.3%	483	14	3%	98.9%	±	5.6%
AMX0002	DRC	85.9576	85.7973	85.9964	85.9%	±	0.1%	0.3%	1	0	3%	497	±	1	2.9%	483	14	3%	99.5%	±	0.1%
AMX0002	DRC	84.1848	87.5634	85.1675	85.6%	±	1.7%	-0.1%	1	0	3%	488	±	10	0.9%	483	14	3%	97.5%	±	2.0%
AMX0002	DRC	82.1112	85.1321	84.9242	84.1%	±	1.7%	-1.9%	1	0	3%	470	±	9	-2.7%	483	14	3%	94.1%	±	1.9%
AMX0003	DRC	83.1485	87.1339	86.8839	85.7%	±	2.2%	0.0%	1	0	1%	489	±	13	-1.8%	498	12	2%	97.8%	±	2.5%
AMX0003	DRC	86.4600	83.0143	85.0963	84.9%	±	1.7%	-1.0%	1	0	1%	498	±	10	-0.1%	498	12	2%	99.5%	±	2.0%
AMX0003	DRC	86.8565	81.7572	85.095	84.6%	±	2.6%	-1.3%	1	0	1%	484	±	15	-2.8%	498	12	2%	96.8%	±	3.0%
AMX0003	DRC	87.3167	85.5883	85.0522	86.0%	±	1.2%	0.3%	1	0	1%	510	±	7	2.3%	498	12	2%	101.9%	±	1.4%
AMX0003	DRC	87.5723	87.4992	87.0906	87.4%	±	0.3%	2.0%	1	0	1%	509	±	2	2.3%	498	12	2%	101.9%	±	0.3%
AMX0004	DRC	86.2525	85.3716	83.9778	85.2%	±	1.1%	-1.3%	1	0	1%	490	±	7	-1.1%	495	11	2%	97.9%	±	1.3%
AMX0004	DRC	86.5330	89.8454	84.3805	86.9%	±	2.8%	0.7%	1	0	1%	504	±	16	1.8%	495	11	2%	100.8%	±	3.2%
AMX0004	DRC	87.5504	86.4525	87.3674	87.1%	±	0.6%	1.0%	1	0	1%	506	±	3	2.2%	495	11	2%	101.1%	±	0.7%
AMX0004	DRC	89.6635	83.1249	87.7753	86.9%	±	3.4%	0.7%	1	0	1%	480	±	19	-3.0%	495	11	2%	96.0%	±	3.7%
AMX0004	DRC	84.0507	85.3651	86.6463	85.4%	±	1.3%	-1.1%	1	0	1%	496	±	8	0.1%	495	11	2%	99.1%	±	1.5%
AMX0005	DRC	77.8297	84.1245	80.4066	80.8%	±	3.2%	-6.8%	1	0	5%	487	±	19	-4.7%	511	23	4%	97.4%	±	3.8%
AMX0005	DRC	88.5439	83.4250	85.5656	85.8%	±	2.6%	-0.9%	1	0	5%	508	±	15	-0.6%	511	23	4%	101.5%	±	3.0%
AMX0005	DRC	88.4809	84.7918	87.0424	86.8%	±	1.9%	0.1%	1	0	5%	502	±	11	-1.7%	511	23	4%	100.5%	±	2.2%
AMX0005	DRC	85.0441	94.1854	83.2395	87.5%	±	5.9%	1.0%	1	0	5%	509	±	34	-0.4%	511	23	4%	101.8%	±	6.8%
AMX0005	DRC	83.5016	96.4738	97.2977	92.4%	±	7.7%	6.6%	1	0	5%	549	±	46	7.4%	511	23	4%	109.8%	±	9.2%
AMX0006	DRC	88.5259	85.0474	87.1195	86.9%	±	1.7%	1.5%	1	0	2%	264	±	5	-0.9%	267	6	2%	105.6%	±	2.1%

FT-IR analysis

AMX0006	DRC	83.6721	87.942	84.2721	85.3%	±	2.3%	-0.3%	1	0	2%	263	±	7	-1.2%	267	6	2%	105.3%	±	2.9%
AMX0006	DRC	83.0873	84.2353	83.6378	83.7%	±	0.6%	-2.3%	1	0	2%	273	±	2	2.3%	267	6	2%	109.1%	±	0.7%
AMX0006	DRC	89.0441	87.972	86.8677	88.0%	±	1.1%	2.8%	1	0	2%	273	±	3	2.3%	267	6	2%	109.1%	±	1.3%
AMX0006	DRC	84.0195	82.0202	86.3732	84.1%	±	2.2%	-1.7%	1	0	2%	260	±	7	-2.4%	267	6	2%	104.0%	±	2.7%
AMX0007	DRC	83.4261	81.4445	82.3278	82.4%	±	1.0%	-0.5%	1	0	1%	498	±	6	1.4%	491	6	1%	99.6%	±	1.2%
AMX0007	DRC	79.0445	82.1737	82.2265	81.1%	±	1.8%	-2.0%	1	0	1%	488	±	11	-0.7%	491	6	1%	97.6%	±	2.2%
AMX0007	DRC	84.0790	79.9911	84.6214	82.9%	±	2.5%	0.1%	1	0	1%	486	±	15	-0.9%	491	6	1%	97.3%	±	3.0%
AMX0007	DRC	83.5535	83.7793	82.7649	83.4%	±	0.5%	0.7%	1	0	1%	485	±	3	-1.2%	491	6	1%	97.1%	±	0.6%
AMX0007	DRC	83.8198	86.5935	82.0373	84.2%	±	2.3%	1.6%	1	0	1%	498	±	14	1.4%	491	6	1%	99.6%	±	2.7%
AMX0008C	GHA	78.5156	82.1948	82.8349	81.2%	±	2.3%	-0.9%	1	0	1%	267	±	15	1.2%	264	4	1%	106.8%	±	3.1%
AMX0008C	GHA	84.3568	83.3286	82.5992	83.4%	±	0.9%	1.8%	1	0	1%	265	±	6	0.5%	264	4	1%	106.1%	±	1.1%
AMX0008C	GHA	81.0486	82.5171	83.7876	82.5%	±	1.4%	0.6%	1	0	1%	265	±	9	0.5%	264	4	1%	106.1%	±	1.8%
AMX0008C	GHA	81.7302	80.483	83.5753	81.9%	±	1.6%	0.0%	1	0	1%	264	±	10	0.2%	264	4	1%	105.8%	±	2.0%
AMX0008C	GHA	81.1791	80.2789	80.3973	80.6%	±	0.5%	-1.6%	1	0	1%	257	±	3	-2.5%	264	4	1%	102.9%	±	0.6%
AMX0009C	GHA	83.5500	82.4786	80.7355	82.3%	±	1.4%	-0.1%	1	0	0%	261	±	9	-1.0%	264	4	2%	104.5%	±	1.8%
AMX0009C	GHA	81.0080	83.3356	82.5076	82.3%	±	1.2%	-0.1%	1	0	0%	261	±	7	-1.3%	264	4	2%	104.2%	±	1.5%
AMX0009C	GHA	80.1553	82.3652	83.6342	82.1%	±	1.8%	-0.3%	1	0	0%	264	±	11	0.0%	264	4	2%	105.6%	±	2.3%
AMX0009C	GHA	81.3128	82.1786	83.3505	82.3%	±	1.0%	-0.1%	1	0	0%	263	±	7	-0.4%	264	4	2%	105.2%	±	1.3%
AMX0009C	GHA	83.1786	82.7809	82.4144	82.8%	±	0.4%	0.6%	1	0	0%	271	±	3	2.7%	264	4	2%	108.4%	±	0.5%
AMX0010C	GHA	82.4325	79.0319	84.1863	81.9%	±	2.6%	0.8%	1	0	1%	267	±	17	0.8%	265	3	1%	106.9%	±	3.4%
AMX0010C	GHA	79.8641	80.215	79.3703	79.8%	±	0.4%	-1.7%	1	0	1%	264	±	3	-0.4%	265	3	1%	105.5%	±	0.6%
AMX0010C	GHA	82.5349	81.1335	82.573	82.1%	±	0.8%	1.1%	1	0	1%	261	±	5	-1.4%	265	3	1%	104.5%	±	1.0%
AMX0010C	GHA	81.6697	80.2603	81.7815	81.2%	±	0.8%	0.0%	1	0	1%	266	±	6	0.2%	265	3	1%	106.3%	±	1.1%
AMX0010C	GHA	79.0439	81.6523	82.4771	81.1%	±	1.8%	-0.2%	1	0	1%	267	±	12	0.8%	265	3	1%	106.9%	±	2.4%
AMX0011C	GHA	83.5788	81.749	81.4718	82.3%	±	1.1%	-0.3%	1	0	1%	267	±	7	1.5%	263	4	1%	106.9%	±	1.5%
AMX0011C	GHA	81.3899	81.8774	80.8875	81.4%	±	0.5%	-1.4%	1	0	1%	258	±	3	-1.9%	263	4	1%	103.3%	±	0.6%
AMX0011C	GHA	84.6315	83.4704	81.1908	83.1%	±	1.8%	0.7%	1	0	1%	263	±	11	-0.1%	263	4	1%	105.2%	±	2.2%
AMX0011C	GHA	81.0956	84.0001	83.3007	82.8%	±	1.5%	0.3%	1	0	1%	262	±	10	-0.5%	263	4	1%	104.8%	±	1.9%
AMX0011C	GHA	82.028	83.3351	83.8174	83.1%	±	0.9%	0.7%	1	0	1%	266	±	6	1.1%	263	4	1%	106.5%	±	1.2%
AMX0012	DRC	87.8959	86.8169	83.8427	86.2%	±	2.1%	-0.4%	1	0	1%	445	±	11	-4.4%	466	22	5%	89.1%	±	2.2%
AMX0012	DRC	85.5892	85.0241	88.9481	87%	±	2%	0.0%	1	0	1%	477	±	12	2.2%	466	22	5%	95.3%	±	2.3%
AMX0012	DRC	89.6929	86.7909	85.5435	87%	±	2%	0.9%	1	0	1%	439	±	11	-5.8%	466	22	5%	87.8%	±	2.1%
AMX0012	DRC	85.3053	86.0702	82.849	85%	±	2%	-2.1%	1	0	1%	486	±	10	4.3%	466	22	5%	97.2%	±	1.9%
AMX0012	DRC	90.9767	88.0688	84.8304	88.0%	±	3.1%	1.6%	1	0	1%	484	±	17	3.8%	466	22	5%	96.8%	±	3.4%
AMX0023A	HAI	81.3183	82.8899	79.4937	81%	±	2%	-0.1%	1	0	1%	501	±	10	15.7%	433	52	12%	100.2%	±	2.1%
AMX0023A	HAI	79.8352	79.6614	80.7152	80%	±	1%	-1.5%	1	0	1%	463	±	3	6.9%	433	52	12%	92.6%	±	0.7%

FT-IR analysis

AMX0023A	HAI	80.8747	83.8015	79.8061	81.5%	±	2.1%	0.2%	1	0	1%	381	±	10	-12.1%	433	52	12%	76.2%	±	1.9%
AMX0023A	HAI	80.9397	82.8259	80.9734	82%	±	1%	0.3%	1	0	1%	383	±	5	-11.6%	433	52	12%	76.6%	±	1.0%
AMX0023A	HAI	78.3225	83.8251	84.6102	82%	±	3%	1.1%	1	0	1%	438	±	18	1.2%	433	52	12%	87.7%	±	3.7%
AMX0023B	HAI	81.9907	83.8859	79.0442	82%	±	2.4%	0.7%	1	0	1%	493	±	15	0.5%	490	11	2%	98.5%	±	2.9%
AMX0023B	HAI	83.0160	78.6814	82.7024	81%	±	2.4%	0.5%	1	0	1%	479	±	14	-2.2%	490	11	2%	95.9%	±	2.8%
AMX0023B	HAI	82.3665	80.7099	78.7598	81%	±	1.8%	-0.6%	1	0	1%	499	±	11	1.9%	490	11	2%	99.9%	±	2.2%
AMX0023B	HAI	81.2560	81.8254	80.8362	81%	±	0.5%	0.3%	1	0	1%	478	±	3	-2.5%	490	11	2%	95.6%	±	0.6%
AMX0023B	HAI	80.8666	80.9046	79.2596	80%	±	0.9%	-0.9%	1	0	1%	502	±	6	2.3%	490	11	2%	100.3%	±	1.2%
AMX0024A	HAI	76.5370	85.8233	77.9075	80%	±	5%	0.2%	1	0	2%	488	±	31	1.7%	480	18	4%	97.7%	±	6.1%
AMX0024A	HAI	78.4438	78.7568	78.4774	79%	±	0%	-1.8%	1	0	2%	480	±	1	0.0%	480	18	4%	96.0%	±	0.2%
AMX0024A	HAI	82.6868	81.2946	80.5206	81.5%	±	1.1%	1.9%	1	0	2%	450	±	6	-6.3%	480	18	4%	90.0%	±	1.2%
AMX0024A	HAI	78.4410	80.5022	78.0741	79%	±	1%	-1.2%	1	0	2%	499	±	8	4.0%	480	18	4%	99.8%	±	1.7%
AMX0024A	HAI	81.075	80.7767	80.2142	81%	±	0%	0.9%	1	0	2%	483	±	3	0.5%	480	18	4%	96.5%	±	0.5%
AMX0024B	HAI	79.2853	82.0875	77.965	80%	±	2%	1.2%	1	0	1%	477	±	13	9.7%	435	75	17%	95.5%	±	2.5%
AMX0024B	HAI	78.3863	78.1005	79.7588	79%	±	1%	-0.1%	1	0	1%	302	±	3	-30.6%	435	75	17%	60.4%	±	0.7%
AMX0024B	HAI	78.4651	79.0226	77.6714	78.4%	±	1%	-0.6%	1	0	1%	454	±	4	4.4%	435	75	17%	90.8%	±	0.8%
AMX0024B	HAI	77.7966	78.2914	78.4859	78%	±	0%	-0.8%	1	0	1%	474	±	2	9.0%	435	75	17%	94.8%	±	0.4%
AMX0024B	HAI	79.9503	79.1308	78.2597	79%	±	1%	0.3%	1	0	1%	468	±	5	7.5%	435	75	17%	93.6%	±	1.0%
AMX0025	IND	83.6422	82.0875	81.6371	82%	±	1.05%	-0.6%	1	0	2%	494	±	6	-1.0%	499	8	2%	98.8%	±	1.3%
AMX0025	IND	81.2396	81.9501	81.9869	82%	±	0.4%	-1.5%	1	0	2%	490	±	3	-1.7%	499	8	2%	98.0%	±	0.5%
AMX0025	IND	80.3601	83.9068	82.939	82%	±	1.8%	-0.7%	1	0	2%	497	±	11	-0.4%	499	8	2%	99.4%	±	2.2%
AMX0025	IND	82.9846	82.5335	82.7663	83%	±	0%	-0.3%	1	0	2%	504	±	1	1.1%	499	8	2%	100.9%	±	0.3%
AMX0025	IND	85.6269	84.1785	86.7018	86%	±	1.3%	3.1%	1	0	2%	509	±	8	2.0%	499	8	2%	101.8%	±	1.5%
AMX0027	PNG	86.0061	81.0852	90.7107	85.9%	±	4.8%	-0.5%	1	0	1%	541	±	30	0.3%	539	12	2%	108.2%	±	6.1%
AMX0027	PNG	86.322	88.0598	89.4591	88%	±	2%	1.9%	1	0	1%	557	±	10	3.2%	539	12	2%	111.3%	±	2.0%
AMX0027	PNG	84.5205	88.4929	85.8746	86.3%	±	2.0%	-0.1%	1	0	1%	541	±	13	0.3%	539	12	2%	108.2%	±	2.5%
AMX0027	PNG	85.1012	83.4059	87.7796	85%	±	2%	-1.1%	1	0	1%	532	±	14	-1.3%	539	12	2%	106.5%	±	2.7%
AMX0027	PNG	84.7704	89.059	84.4958	86.1%	±	2.6%	-0.3%	1	0	1%	526	±	16	-2.5%	539	12	2%	105.2%	±	3.1%
AMX0028	ETH	79.662	80.961	81.8742	80.8%	±	1.1%	0.2%	1	0	1%	484	±	7	1.8%	476	15	3%	96.9%	±	1.3%
AMX0028	ETH	80.8763	79.2761	80.5102	80%	±	1%	-0.6%	1	0	1%	490	±	5	2.9%	476	15	3%	97.9%	±	1.0%
AMX0028	ETH	80.6655	80.0372	79.0107	79.9%	±	0.8%	-1.0%	1	0	1%	452	±	5	-5.1%	476	15	3%	90.4%	±	0.9%
AMX0028	ETH	80.8966	80.4248	81.5545	81%	±	1%	0.3%	1	0	1%	478	±	3	0.6%	476	15	3%	95.7%	±	0.7%
AMX0028	ETH	82.452	81.591	80.4399	81.5%	±	1.0%	1.0%	1	0	1%	475	±	6	-0.2%	476	15	3%	95.0%	±	1.2%
AMX0029A	ETH	79.3347	79.8215	79.9179	79.7%	±	0.3%	0.7%	1	0	1%	458	±	2	1.0%	453	4	1%	91.5%	±	0.4%
AMX0029A	ETH	77.5033	78.6078	78.1682	78.1%	±	0.6%	-1.3%	1	0	1%	448	±	3	-1.0%	453	4	1%	89.7%	±	0.6%
AMX0029A	ETH	79.4641	79.5642	78.5292	79.19%	±	0.57%	0.1%	1	0	1%	452	±	3	-0.3%	453	4	1%	90.4%	±	0.7%

FT-IR analysis

AMX0029A	ETH	78.9751	79.5402	77.4317	78.6%	±	1.1%	-0.6%	1	0	1%	450	±	6	-0.6%	453	4	1%	90.0%	±	1.2%
AMX0029A	ETH	82.6995	76.846	80.7144	80.09%	±	2.98%	1.2%	1	0	1%	457	±	17	0.9%	453	4	1%	91.4%	±	3.4%
AMX0029B	ETH	78.0501	77.2087	77.7007	77.7%	±	0.4%	-0.9%	1	0	1%	445	±	2	-1.4%	451	5	1%	89.0%	±	0.5%
AMX0029B	ETH	76.5407	77.6424	84.0732	79%	±	4%	1.4%	1	0	1%	457	±	23	1.3%	451	5	1%	91.4%	±	4.7%
AMX0029B	ETH	77.7607	77.5184	77.5726	77.6%	±	0.1%	-0.9%	1	0	1%	449	±	1	-0.5%	451	5	1%	89.8%	±	0.1%
AMX0029B	ETH	78.4305	78.6512	78.7701	79%	±	0%	0.3%	1	0	1%	454	±	1	0.5%	451	5	1%	90.7%	±	0.2%
AMX0029B	ETH	77.8189	79.5426	77.962	78%	±	1%	0.1%	1	0	1%	451	±	6	0.0%	451	5	1%	90.3%	±	1.1%
AMX0031A	SLE	85.2186	84.4905	79.9496	83.2%	±	2.9%	2.2%	1	0	1%	254	±	9	-0.4%	255	1	1%	101.5%	±	3.5%
AMX0031A	SLE	80.2584	80.5709	82.1159	81.0%	±	1.0%	-0.5%	1	0	1%	253	±	3	-0.6%	255	1	1%	101.3%	±	1.2%
AMX0031A	SLE	80.0701	82.5251	81.7246	81.4%	±	1.3%	0.0%	1	0	1%	254	±	4	-0.2%	255	1	1%	101.7%	±	1.6%
AMX0031A	SLE	80.1229	81.2469	80.255	81%	±	1%	-1.1%	1	0	1%	256	±	2	0.6%	255	1	1%	102.5%	±	0.8%
AMX0031A	SLE	81.2418	80.3909	80.8201	80.8%	±	0.4%	-0.7%	1	0	1%	256	±	1	0.6%	255	1	1%	102.5%	±	0.5%
AMX0031B	SLE	79.0783	81.4097	82.233	80.9%	±	1.6%	-1.2%	1	0	1%	258	±	5	0.3%	257	6	2%	103.3%	±	2.1%
AMX0031B	SLE	81.9527	83.1162	82.0424	82.4%	±	0.6%	0.6%	1	0	1%	266	±	2	3.3%	257	6	2%	106.4%	±	0.8%
AMX0031B	SLE	81.4318	81.7535	82.1556	81.8%	±	0.4%	-0.1%	1	0	1%	251	±	1	-2.6%	257	6	2%	100.3%	±	0.4%
AMX0031B	SLE	82.7743	83.932	80.7325	82%	±	2%	0.8%	1	0	1%	259	±	5	0.6%	257	6	2%	103.5%	±	2.0%
AMX0031B	SLE	81.3851	84.3113	79.5391	81.7%	±	2.4%	-0.1%	1	0	1%	253	±	7	-1.7%	257	6	2%	101.2%	±	3.0%
AMX0031C	SLE	81.5701	83.125	80.2609	81.7%	±	1.4%	-0.5%	1	0	2%	258	±	5	1.5%	254	6	2%	103.2%	±	1.8%
AMX0031C	SLE	84.9493	84.414	80.6752	83.3%	±	2.3%	1.5%	1	0	2%	259	±	7	1.9%	254	6	2%	103.6%	±	2.9%
AMX0031C	SLE	82.3308	83.5778	84.6026	83.5%	±	1.1%	1.7%	1	0	2%	256	±	3	0.6%	254	6	2%	102.2%	±	1.4%
AMX0031C	SLE	81.5202	81.7699	78.5998	81%	±	2%	-1.8%	1	0	2%	244	±	5	-3.9%	254	6	2%	97.7%	±	2.1%
AMX0031C	SLE	80.3388	82.2531	81.2226	81.3%	±	1.0%	-1.0%	1	0	2%	254	±	3	0.0%	254	6	2%	101.7%	±	1.2%
AMX0032	SLE	81.788	79.653	80.4238	80.6%	±	1.1%	-1.9%	1	0	2%	474	±	6	-2.0%	483	17	3%	94.7%	±	1.3%
AMX0032	SLE	79.0768	77.7418	81.8263	79.5%	±	2.1%	-3.2%	1	0	2%	459	±	12	-5.0%	483	17	3%	91.8%	±	2.4%
AMX0032	SLE	81.156	83.9993	85.5306	83.6%	±	2.2%	1.7%	1	0	2%	494	±	13	2.2%	483	17	3%	98.8%	±	2.6%
AMX0032	SLE	82.1765	83.4879	85.1035	84%	±	1%	1.7%	1	0	2%	499	±	9	3.3%	483	17	3%	99.8%	±	1.8%
AMX0032	SLE	83.2471	84.7237	82.691	83.6%	±	1.1%	1.7%	1	0	2%	491	±	6	1.5%	483	17	3%	98.1%	±	1.2%
AMX0033A	SLE	84.5842	84.8214	81.9128	83.8%	±	1.6%	0.7%	1	0	1%	502	±	10	0.6%	499	6	1%	100.3%	±	1.9%
AMX0033A	SLE	81.7639	84.2571	81.1403	82.4%	±	1.6%	-0.9%	1	0	1%	496	±	10	-0.6%	499	6	1%	99.2%	±	2.0%
AMX0033A	SLE	82.5252	84.8521	87.1278	84.8%	±	2.3%	2.0%	1	0	1%	504	±	14	1.0%	499	6	1%	100.7%	±	2.7%
AMX0033A	SLE	83.056	82.9915	83.1311	83%	±	0%	-0.1%	1	0	1%	503	±	0	0.8%	499	6	1%	100.5%	±	0.1%
AMX0033A	SLE	80.2238	83.4986	81.6422	81.8%	±	1.6%	-1.7%	1	0	1%	490	±	10	-1.7%	499	6	1%	98.0%	±	2.0%
AMX0033B	SLE	84.9906	82.4748	82.448	83.3%	±	1.5%	0.4%	1	0	1%	501	±	9	-0.6%	504	4	1%	100.2%	±	1.8%
AMX0033B	SLE	84.1625	83.0032	83.5919	83.6%	±	0.6%	0.7%	1	0	1%	504	±	3	0.1%	504	4	1%	100.9%	±	0.7%
AMX0033B	SLE	81.354	83.5402	83.5757	82.8%	±	1.3%	-0.2%	1	0	1%	510	±	8	1.2%	504	4	1%	101.9%	±	1.6%
AMX0033B	SLE	83.5781	85.6511	83.4227	84%	±	1%	1.5%	1	0	1%	503	±	7	-0.2%	504	4	1%	100.6%	±	1.5%

FT-IR analysis

AMX0033B	SLE	79.1329	81.5767	82.5508	81.1% ±	1.8%	-2.3%	1	0	1%	501 ±	11	-0.6%	504	4	1%	100.2% ±	2.2%
AMX0033C	SLE	80.0677	80.5443	80.0142	80.2% ±	0.3%	-1.5%	1	0	2%	477 ±	2	-2.7%	490	13	3%	95.4% ±	0.3%
AMX0033C	SLE	79.8343	83.023	81.4859	81.4% ±	1.6%	0.0%	1	0	2%	481 ±	9	-1.8%	490	13	3%	96.3% ±	1.9%
AMX0033C	SLE	83.1876	83.3083	85.145	83.9% ±	1.1%	3.0%	1	0	2%	510 ±	7	4.0%	490	13	3%	101.9% ±	1.3%
AMX0033C	SLE	83.8665	81.5382	78.5747	81% ±	3%	-0.2%	1	0	2%	494 ±	16	0.9%	490	13	3%	98.9% ±	3.2%
AMX0033C	SLE	78.9596	81.6583	80.7813	80.5% ±	1.4%	-1.2%	1	0	2%	488 ±	8	-0.5%	490	13	3%	97.5% ±	1.7%
AMX0033D	SLE	82.286	82.6957	93.0381	86.0% ±	6.1%	4.1%	1	0	3%	526 ±	37	5.1%	501	15	3%	105.2% ±	7.5%
AMX0033D	SLE	82.4281	81.2066	86.922	83.5% ±	3.0%	1.1%	1	0	3%	499 ±	18	-0.3%	501	15	3%	99.8% ±	3.6%
AMX0033D	SLE	82.6869	81.0529	81.3856	81.7% ±	0.9%	-1.1%	1	0	3%	496 ±	5	-1.0%	501	15	3%	99.1% ±	1.0%
AMX0033D	SLE	81.8944	80.8103	81.0795	81% ±	1%	-1.6%	1	0	3%	496 ±	3	-1.0%	501	15	3%	99.2% ±	0.7%
AMX0033D	SLE	80.5974	80.0951	80.8392	80.5% ±	0.4%	-2.5%	1	0	3%	486 ±	2	-2.8%	501	15	3%	97.3% ±	0.5%
AMX0034A	SLE	82.0677	83.621	79.7616	81.8% ±	1.9%	-1.5%	1	0	2%	489 ±	12	-2.3%	501	12	2%	97.8% ±	2.3%
AMX0034A	SLE	81.6469	83.6652	81.0383	82.1% ±	1.4%	-1.1%	1	0	2%	495 ±	8	-1.2%	501	12	2%	98.9% ±	1.7%
AMX0034A	SLE	78.9037	84.1378	82.5923	81.9% ±	2.7%	-1.4%	1	0	2%	492 ±	16	-1.7%	501	12	2%	98.5% ±	3.2%
AMX0034A	SLE	84.5277	84.3713	82.9353	84% ±	1%	1.1%	1	0	2%	513 ±	5	2.4%	501	12	2%	102.5% ±	1.1%
AMX0034A	SLE	84.5531	86.4242	85.7076	85.6% ±	0.9%	3.0%	1	0	2%	515 ±	6	2.8%	501	12	2%	102.9% ±	1.1%
AMX0034B	SLE	82.8407	81.4612	81.0837	81.8% ±	0.9%	-1.5%	1	0	1%	490 ±	6	-1.2%	496	4	1%	98.0% ±	1.1%
AMX0034B	SLE	83.5476	81.6921	82.6692	82.6% ±	0.9%	-0.5%	1	0	1%	496 ±	6	-0.1%	496	4	1%	99.2% ±	1.1%
AMX0034B	SLE	82.0393	82.9156	85.7973	83.6% ±	2.0%	0.7%	1	0	1%	501 ±	12	1.0%	496	4	1%	100.2% ±	2.4%
AMX0034B	SLE	85.2656	86.8106	82.5044	85% ±	2%	2.2%	1	0	1%	499 ±	13	0.6%	496	4	1%	99.9% ±	2.6%
AMX0034B	SLE	84.0402	82.0019	80.9002	82.3% ±	1.6%	-0.9%	1	0	1%	495 ±	10	-0.2%	496	4	1%	99.0% ±	1.9%
AMX0035A	SLE	82.2947	84.4559	83.9387	83.6% ±	1.1%	0.1%	1	0	1%	481 ±	6	-1.1%	486	9	2%	96.2% ±	1.3%
AMX0035A	SLE	83.4986	82.3427	84.7573	83.5% ±	1.2%	0.1%	1	0	1%	485 ±	7	-0.3%	486	9	2%	97.0% ±	1.4%
AMX0035A	SLE	83.1136	82.9811	86.3976	84.2% ±	1.9%	0.8%	1	0	1%	476 ±	11	-2.2%	486	9	2%	95.1% ±	2.2%
AMX0035A	SLE	82.8489	82.7363	83.5088	83% ±	0%	-0.5%	1	0	1%	497 ±	2	2.2%	486	9	2%	99.4% ±	0.5%
AMX0035A	SLE	82.8311	81.9167	84.3228	83.0% ±	1.2%	-0.5%	1	0	1%	493 ±	7	1.4%	486	9	2%	98.6% ±	1.4%
AMX0035B	SLE	83.5854	82.9517	84.8501	83.8% ±	1.0%	-0.5%	1	0	1%	477 ±	6	-1.8%	486	9	2%	95.4% ±	1.1%
AMX0035B	SLE	83.0558	87.083	85.4707	85.2% ±	2.0%	1.2%	1	0	1%	489 ±	12	0.7%	486	9	2%	97.9% ±	2.3%
AMX0035B	SLE	83.6534	84.6724	82.4327	83.6% ±	1.1%	-0.7%	1	0	1%	499 ±	7	2.7%	486	9	2%	99.8% ±	1.3%
AMX0035B	SLE	84.4608	86.5296	80.0013	84% ±	3%	-0.6%	1	0	1%	482 ±	19	-0.7%	486	9	2%	96.5% ±	3.8%
AMX0035B	SLE	81.9632	87.1186	84.8839	84.7% ±	2.6%	0.6%	1	0	1%	482 ±	15	-0.8%	486	9	2%	96.4% ±	2.9%
AMX0036A	SLE	84.7152	87.8317	82.2002	84.9% ±	2.8%	-0.6%	1	0	2%	252 ±	8	2.6%	246	8	3%	100.9% ±	3.4%
AMX0036A	SLE	84.5965	83.9151	83.3535	84.0% ±	0.6%	-1.8%	1	0	2%	243 ±	2	-1.2%	246	8	3%	97.2% ±	0.7%
AMX0036A	SLE	83.6642	97.9411	82.1896	87.9% ±	8.7%	2.9%	1	0	2%	255 ±	25	3.8%	246	8	3%	102.1% ±	10.1%
AMX0036A	SLE	86.1757	85.5427	90.5189	87% ±	3%	2.3%	1	0	2%	244 ±	8	-0.9%	246	8	3%	97.5% ±	3.0%
AMX0036A	SLE	85.1638	82.9605	81.173	83.1% ±	2.0%	-2.8%	1	0	2%	235 ±	6	-4.3%	246	8	3%	94.1% ±	2.3%

FT-IR analysis

AMX0036B	SLE	80.7603	82.3526	82.9736	82.0% ±	1.1%	-0.4%	1	0	1%	206 ±	3	-13.9%	239	21	9%	82.3% ±	1.1%
AMX0036B	SLE	85.7963	81.346	82.801	83.3% ±	2.3%	1.1%	1	0	1%	242 ±	7	1.3%	239	21	9%	96.8% ±	2.6%
AMX0036B	SLE	81.7351	81.7431	82.2013	81.9% ±	0.3%	-0.6%	1	0	1%	253 ±	1	5.8%	239	21	9%	101.1% ±	0.3%
AMX0036B	SLE	82.1785	82.8337	81.2514	82% ±	1%	-0.4%	1	0	1%	235 ±	2	-1.6%	239	21	9%	94.1% ±	0.9%
AMX0036B	SLE	84.2218	81.4751	82.2594	82.7% ±	1.4%	0.3%	1	0	1%	259 ±	4	8.3%	239	21	9%	103.5% ±	1.8%
AMX0037A	SLE	82.8144	81.2482	81.7573	81.9% ±	0.8%	-0.3%	1	0	1%	475 ±	5	-1.4%	482	17	3%	95.0% ±	0.9%
AMX0037A	SLE	82.9339	81.028	86.021	83.3% ±	2.5%	1.3%	1	0	1%	457 ±	14	-5.2%	482	17	3%	91.3% ±	2.8%
AMX0037A	SLE	79.8288	83.0934	84.1017	82.3% ±	2.2%	0.1%	1	0	1%	501 ±	14	4.0%	482	17	3%	100.2% ±	2.7%
AMX0037A	SLE	85.4404	81.704	82.0052	83% ±	2%	1.0%	1	0	1%	489 ±	12	1.6%	482	17	3%	97.9% ±	2.4%
AMX0037A	SLE	81.2902	79.1986	80.886	80.5% ±	1.1%	-2.1%	1	0	1%	486 ±	7	0.8%	482	17	3%	97.1% ±	1.3%
AMX0038A	SLE	81.6513	82.2265	79.6808	81.2% ±	1.3%	0.6%	1	0	1%	234 ±	4	0.3%	233	3	1%	93.6% ±	1.5%
AMX0038A	SLE	80.1071	79.467	79.9793	79.9% ±	0.3%	-1.0%	1	0	1%	234 ±	1	0.3%	233	3	1%	93.6% ±	0.4%
AMX0038A	SLE	82.2911	82.9695	81.9672	82.4% ±	0.5%	2.1%	1	0	1%	238 ±	1	1.9%	233	3	1%	95.2% ±	0.6%
AMX0038A	SLE	79.4009	79.8571	81.5248	80% ±	1%	-0.5%	1	0	1%	230 ±	3	-1.5%	233	3	1%	91.9% ±	1.3%
AMX0038A	SLE	80.6856	80.1244	78.3313	79.7% ±	1.2%	-1.2%	1	0	1%	231 ±	4	-1.0%	233	3	1%	92.5% ±	1.4%
AMX0038B	SLE	79.7662	77.8075	79.8823	79.2% ±	1.2%	-0.2%	1	0	0%	228 ±	3	0.3%	227	2	1%	91.2% ±	1.3%
AMX0038B	SLE	79.7063	78.3668	80.1917	79.4% ±	0.9%	0.2%	1	0	0%	229 ±	3	0.9%	227	2	1%	91.7% ±	1.1%
AMX0038B	SLE	80.0251	79.0199	79.4496	79.5% ±	0.5%	0.3%	1	0	0%	227 ±	1	0.1%	227	2	1%	91.0% ±	0.6%
AMX0038B	SLE	76.3366	81.6457	79.5891	79% ±	3%	-0.1%	1	0	0%	227 ±	8	-0.2%	227	2	1%	90.7% ±	3.1%
AMX0038B	SLE	79.1342	80.113	78.3873	79.2% ±	0.9%	-0.1%	1	0	0%	224 ±	2	-1.2%	227	2	1%	89.8% ±	1.0%
AMX0039A	SLE	79.819	80.5332	81.3825	80.6% ±	0.8%	-0.1%	1	0	1%	497 ±	5	2.1%	486	10	2%	99.3% ±	1.0%
AMX0039A	SLE	82.7776	80.3869	78.5582	80.6% ±	2.1%	-0.1%	1	0	1%	480 ±	13	-1.2%	486	10	2%	96.1% ±	2.5%
AMX0039A	SLE	81.4602	78.9473	79.3948	79.9% ±	1.3%	-0.9%	1	0	1%	477 ±	8	-2.0%	486	10	2%	95.3% ±	1.6%
AMX0039A	SLE	79.5755	80.1815	81.11	80% ±	1%	-0.4%	1	0	1%	480 ±	5	-1.2%	486	10	2%	96.1% ±	0.9%
AMX0039A	SLE	80.6024	82.2891	82.6836	81.9% ±	1.1%	1.5%	1	0	1%	497 ±	7	2.2%	486	10	2%	99.4% ±	1.3%
AMX0039B	SLE	83.4362	81.7603	78.3564	81.2% ±	2.6%	-0.7%	1	0	2%	496 ±	16	-0.3%	498	6	1%	99.3% ±	3.2%
AMX0039B	SLE	86.1742	81.9687	82.6779	83.6% ±	2.3%	2.3%	1	0	2%	503 ±	14	1.1%	498	6	1%	100.7% ±	2.7%
AMX0039B	SLE	83.0551	80.2719	83.1452	82.2% ±	1.6%	0.5%	1	0	2%	490 ±	10	-1.6%	498	6	1%	97.9% ±	1.9%
AMX0039B	SLE	80.0783	79.5215	79.9103	80% ±	0%	-2.3%	1	0	2%	495 ±	2	-0.5%	498	6	1%	99.1% ±	0.4%
AMX0039B	SLE	81.6821	82.7086	81.5869	82.0% ±	0.6%	0.3%	1	0	2%	504 ±	4	1.2%	498	6	1%	100.8% ±	0.8%
AMX0040A	SLE	80.9839	81.7286	80.6884	81.1% ±	0.5%	1.4%	1	0	1%	486 ±	3	1.4%	479	7	1%	97.1% ±	0.6%
AMX0040A	SLE	79.4058	79.9457	79.3225	79.6% ±	0.3%	-0.6%	1	0	1%	470 ±	2	-1.9%	479	7	1%	94.0% ±	0.4%
AMX0040A	SLE	79.5153	79.8438	79.0739	79.5% ±	0.4%	-0.7%	1	0	1%	473 ±	2	-1.2%	479	7	1%	94.6% ±	0.5%
AMX0040A	SLE	79.1081	81.4728	79.6688	80% ±	1%	0.1%	1	0	1%	481 ±	7	0.4%	479	7	1%	96.2% ±	1.5%
AMX0040A	SLE	79.1588	79.679	80.684	79.8% ±	0.8%	-0.2%	1	0	1%	485 ±	5	1.3%	479	7	1%	97.0% ±	0.9%

FT-IR analysis

AMX0040B	SLE	77.1413	75.8958	77.8043	76.9%	±	1.0%	-2.9%	1	0	2%	445	±	6	-4.9%	469	13	3%	89.1%	±	1.1%
AMX0040B	SLE	80.3613	78.8354	80.8564	80.0%	±	1.1%	1.0%	1	0	2%	473	±	6	1.0%	469	13	3%	94.7%	±	1.2%
AMX0040B	SLE	78.8856	80.0715	81.7479	80.2%	±	1.4%	1.3%	1	0	2%	480	±	9	2.3%	469	13	3%	95.9%	±	1.7%
AMX0040B	SLE	80.2742	79.9827	79.0164	80%	±	1%	0.7%	1	0	2%	469	±	4	0.1%	469	13	3%	93.8%	±	0.8%
AMX0040B	SLE	80.0809	78.5416	79.1161	79.2%	±	0.8%	0.0%	1	0	2%	476	±	5	1.5%	469	13	3%	95.1%	±	0.9%
AMX0041	GHA	80.8991	82.0208	79.6116	80.8%	±	1.2%	1.1%	1	0	2%	486	±	7	2.0%	476	12	2%	97.2%	±	1.4%
AMX0041	GHA	83.1399	80.9016	79.3926	81.1%	±	1.9%	1.4%	1	0	2%	481	±	11	0.9%	476	12	2%	96.2%	±	2.2%
AMX0041	GHA	80.0747	77.559	78.1075	78.6%	±	1.3%	-1.8%	1	0	2%	463	±	8	-2.8%	476	12	2%	92.6%	±	1.6%
AMX0041	GHA	78.2765	78.5282	79.0118	79%	±	0%	-1.7%	1	0	2%	465	±	2	-2.5%	476	12	2%	92.9%	±	0.4%
AMX0041	GHA	82.3378	79.9785	80.0966	80.8%	±	1.3%	1.0%	1	0	2%	487	±	8	2.3%	476	12	2%	97.4%	±	1.6%
AMX0042	GHA	79.0385	80.0349	80.5814	79.9%	±	0.8%	-1.1%	1	0	1%	469	±	5	-1.9%	478	6	1%	93.8%	±	0.9%
AMX0042	GHA	82.7035	81.1835	79.2582	81.0%	±	1.7%	0.4%	1	0	1%	483	±	10	1.0%	478	6	1%	96.5%	±	2.1%
AMX0042	GHA	81.2618	81.4105	82.44	81.7%	±	0.6%	1.2%	1	0	1%	484	±	4	1.2%	478	6	1%	96.7%	±	0.8%
AMX0042	GHA	80.6793	79.9565	79.8393	80%	±	0%	-0.7%	1	0	1%	475	±	3	-0.6%	478	6	1%	95.0%	±	0.5%
AMX0042	GHA	81.7201	79.444	81.4432	80.9%	±	1.2%	0.2%	1	0	1%	479	±	7	0.2%	478	6	1%	95.8%	±	1.5%
AMX0043	GHA	81.2894	80.8611	85.6878	82.6%	±	2.7%	1.1%	1	0	1%	483	±	16	0.0%	483	7	1%	96.7%	±	3.1%
AMX0043	GHA	82.3179	80.9294	82.144	81.8%	±	0.8%	0.1%	1	0	1%	492	±	5	1.8%	483	7	1%	98.4%	±	0.9%
AMX0043	GHA	82.7991	80.7235	83.7681	82.4%	±	1.6%	0.9%	1	0	1%	488	±	9	0.9%	483	7	1%	97.5%	±	1.8%
AMX0043	GHA	81.1948	79.5485	82.6332	81%	±	2%	-0.7%	1	0	1%	480	±	9	-0.7%	483	7	1%	96.0%	±	1.8%
AMX0043	GHA	80.2256	81.0931	80.167	80.5%	±	0.5%	-1.5%	1	0	1%	474	±	3	-2.0%	483	7	1%	94.8%	±	0.6%
AMX0044	GHA	77.4069	78.2584	80.0871	78.6%	±	1.4%	-1.2%	1	0	1%	474	±	8	-0.6%	476	3	1%	94.7%	±	1.7%
AMX0044	GHA	78.1062	79.6831	80.3372	79.4%	±	1.1%	-0.2%	1	0	1%	475	±	7	-0.3%	476	3	1%	95.0%	±	1.4%
AMX0044	GHA	80.3645	79.4565	79.5528	79.8%	±	0.5%	0.3%	1	0	1%	474	±	3	-0.4%	476	3	1%	94.9%	±	0.6%
AMX0044	GHA	79.6888	80.4315	79.795	80%	±	0%	0.6%	1	0	1%	477	±	2	0.2%	476	3	1%	95.4%	±	0.5%
AMX0044	GHA	78.49	81.6811	79.629	79.9%	±	1.6%	0.5%	1	0	1%	481	±	10	1.0%	476	3	1%	96.2%	±	1.9%
AMX0045	GHA	79.1779	79.7576	79.5663	79.5%	±	0.3%	-0.6%	1	0	1%	462	±	2	2.7%	449	10	2%	92.3%	±	0.3%
AMX0045	GHA	81.8809	80.7551	80.928	81.2%	±	0.6%	1.5%	1	0	1%	455	±	3	1.2%	449	10	2%	90.9%	±	0.7%
AMX0045	GHA	79.9007	79.3045	78.4051	79.2%	±	0.8%	-0.9%	1	0	1%	454	±	4	0.9%	449	10	2%	90.7%	±	0.9%
AMX0045	GHA	79.7796	79.1252	80.5236	80%	±	1%	-0.2%	1	0	1%	438	±	4	-2.5%	449	10	2%	87.6%	±	0.8%
AMX0045	GHA	80.2439	80.8147	79.1932	80.1%	±	0.8%	0.2%	1	0	1%	439	±	5	-2.4%	449	10	2%	87.7%	±	0.9%
AMX0046	GHA	76.8868	76.7507	76.7426	76.8%	±	0.1%	-0.5%	1	0	0%	477	±	1	-1.7%	485	11	2%	95.4%	±	0.1%
AMX0046	GHA	76.5584	76.9922	78.3871	77.3%	±	1.0%	0.2%	1	0	0%	475	±	6	-2.1%	485	11	2%	95.0%	±	1.2%
AMX0046	GHA	77.235	78.5429	76.4732	77.4%	±	1.0%	0.3%	1	0	0%	495	±	7	2.0%	485	11	2%	99.0%	±	1.3%
AMX0046	GHA	77.1652	77.2569	76.5009	77%	±	0%	-0.2%	1	0	0%	480	±	3	-1.2%	485	11	2%	95.9%	±	0.5%
AMX0046	GHA	77.2637	76.5108	78.1747	77.3%	±	0.8%	0.2%	1	0	0%	500	±	5	2.9%	485	11	2%	99.9%	±	1.1%
AMX0047	GHA	79.6112	79.566	80.7192	80.0%	±	0.7%	-0.9%	1	0	1%	441	±	4	-6.7%	473	19	4%	88.2%	±	0.7%

FT-IR analysis

AMX0047	GHA	78.1627	81.5979	81.6637	80.5%	±	2.0%	-0.3%	1	0	1%	472	±	12	-0.1%	473	19	4%	94.4%	±	2.3%
AMX0047	GHA	80.0152	78.7847	80.131	79.6%	±	0.7%	-1.3%	1	0	1%	478	±	4	1.1%	473	19	4%	95.6%	±	0.9%
AMX0047	GHA	80.6468	81.5961	80.5735	81%	±	1%	0.3%	1	0	1%	483	±	3	2.1%	473	19	4%	96.5%	±	0.7%
AMX0047	GHA	82.8787	82.104	82.5395	82.5%	±	0.4%	2.2%	1	0	1%	490	±	2	3.7%	473	19	4%	98.0%	±	0.5%
AMX0048A	HAI	82.3835	81.2267	81.0328	81.5%	±	0.7%	0.8%	1	0	1%	474	±	4	-1.1%	479	5	1%	94.8%	±	0.8%
AMX0048A	HAI	83.1727	81.0621	81.8902	82.0%	±	1.1%	1.4%	1	0	1%	475	±	6	-0.8%	479	5	1%	95.1%	±	1.2%
AMX0048A	HAI	79.3824	80.1789	80.4418	80%	±	1%	-1.1%	1	0	1%	485	±	3	1.3%	479	5	1%	97.0%	±	0.7%
AMX0048A	HAI	80.2272	79.1041	80.4849	79.9%	±	0.7%	-1.2%	1	0	1%	482	±	4	0.6%	479	5	1%	96.4%	±	0.9%
AMX0048B	HAI	80.91	82.3145	80.4685	81.2%	±	1.0%	-0.4%	1	0	1%	482	±	6	1.1%	476	12	2%	96.4%	±	1.1%
AMX0048B	HAI	81.1114	81.3153	82.7643	81.7%	±	0.9%	0.2%	1	0	1%	479	±	5	0.6%	476	12	2%	95.8%	±	1.1%
AMX0048B	HAI	81.1581	81.5022	80.308	81.0%	±	0.6%	-0.7%	1	0	1%	488	±	4	2.5%	476	12	2%	97.7%	±	0.7%
AMX0048B	HAI	81.1576	80.168	83.0903	81%	±	1%	-0.1%	1	0	1%	457	±	8	-4.0%	476	12	2%	91.5%	±	1.7%
AMX0048B	HAI	80.6501	84.1608	82.6785	82.5%	±	1.8%	1.1%	1	0	1%	475	±	10	-0.3%	476	12	2%	95.0%	±	2.0%
AMX0048C	HAI	80.4666	81.6631	80.4397	80.9%	±	0.7%	1.3%	1	0	1%	472	±	4	0.6%	469	7	1%	94.4%	±	0.8%
AMX0048C	HAI	81.3004	78.1811	78.3789	79.3%	±	1.7%	-0.7%	1	0	1%	464	±	10	-1.2%	469	7	1%	92.7%	±	2.0%
AMX0048C	HAI	81.9193	78.0845	78.7265	79.6%	±	2.1%	-0.3%	1	0	1%	466	±	12	-0.6%	469	7	1%	93.3%	±	2.4%
AMX0048C	HAI	79.9372	80.3058	80.0806	80%	±	0%	0.3%	1	0	1%	479	±	1	2.2%	469	7	1%	95.9%	±	0.2%
AMX0048C	HAI	78.6919	78.7375	80.6416	79.4%	±	1.1%	-0.6%	1	0	1%	464	±	7	-1.1%	469	7	1%	92.8%	±	1.3%
AMX0050A	DRC	50.9453	38.7741	45.2428	45.0%	±	6.1%	-3.8%	0	0	7%	267	±	36	4.4%	255	21	8%	106.6%	±	14.4%
AMX0050A	DRC	44.4027	42.7493	38.6484	41.9%	±	3.0%	-10.3%	0	0	7%	223	±	16	-12.6%	255	21	8%	89.2%	±	6.3%
AMX0050A	DRC	47.3109	50.5024	48.3184	48.7%	±	1.6%	4.2%	0	0	7%	244	±	8	-4.5%	255	21	8%	97.5%	±	3.3%
AMX0050A	DRC	57.1801	43.2933	46.5177	49%	±	7%	4.8%	0	0	7%	271	±	40	6.2%	255	21	8%	108.5%	±	16.1%
AMX0050A	DRC	44.9904	52.8012	49.7078	49.2%	±	3.9%	5.1%	0	0	7%	272	±	22	6.6%	255	21	8%	108.9%	±	8.7%
AMX0050B	DRC	54.5254	54.461	48.1687	52.4%	±	3.7%	10.6%	0	0	8%	283	±	20	11.5%	254	27	11%	113.3%	±	7.9%
AMX0050B	DRC	45.9601	43.8522	39.8601	43.2%	±	3.1%	-8.8%	0	0	8%	218	±	16	-14.1%	254	27	11%	87.2%	±	6.3%
AMX0050B	DRC	43.4185	46.242	49.5214	46.4%	±	3.1%	-2.1%	0	0	8%	241	±	16	-4.9%	254	27	11%	96.6%	±	6.4%
AMX0050B	DRC	48.9889	44.4565	55.9162	50%	±	6%	5.1%	0	0	8%	279	±	32	10.0%	254	27	11%	111.6%	±	12.9%
AMX0050B	DRC	50.0602	40.7502	44.5528	45.1%	±	4.7%	-4.8%	0	0	8%	247	±	26	-2.6%	254	27	11%	98.9%	±	10.3%
AMX0051A	DRC	80.6332	81.1803	82.1355	81.3%	±	0.8%	0.7%	1	0	1%	428	±	4	-9.9%	475	27	6%	85.6%	±	0.8%
AMX0051A	DRC	80.7251	80.3986	79.6111	80.2%	±	0.6%	-0.7%	1	0	1%	486	±	3	2.3%	475	27	6%	97.2%	±	0.7%
AMX0051A	DRC	82.6534	79.9515	81.813	81.5%	±	1.4%	0.9%	1	0	1%	492	±	8	3.5%	475	27	6%	98.4%	±	1.7%
AMX0051A	DRC	81.5697	81.5075	79.2595	81%	±	1%	0.0%	1	0	1%	488	±	8	2.7%	475	27	6%	97.5%	±	1.6%
AMX0051A	DRC	79.9157	79.1045	81.2816	80.1%	±	1.1%	-0.8%	1	0	1%	482	±	7	1.4%	475	27	6%	96.4%	±	1.3%
AMX0051B	DRC	82.1331	80.2367	81.2009	81.2%	±	0.9%	-0.1%	1	0	1%	491	±	6	0.2%	490	4	1%	98.3%	±	1.1%
AMX0051B	DRC	83.4266	80.4513	80.3803	81.4%	±	1.7%	0.2%	1	0	1%	491	±	10	0.2%	490	4	1%	98.3%	±	2.1%
AMX0051B	DRC	79.4843	81.7883	80.2359	80.5%	±	1.2%	-1.0%	1	0	1%	485	±	7	-1.0%	490	4	1%	97.1%	±	1.4%

FT-IR analysis

AMX0051B	DRC	82.541	81.9262	81.2988	82%	±	1%	0.8%	1	0	1%	496	±	4	1.2%	490	4	1%	99.2%	±	0.8%
AMX0051B	DRC	80.9017	79.6026	83.7235	81.4%	±	2.1%	0.1%	1	0	1%	488	±	13	-0.5%	490	4	1%	97.6%	±	2.5%

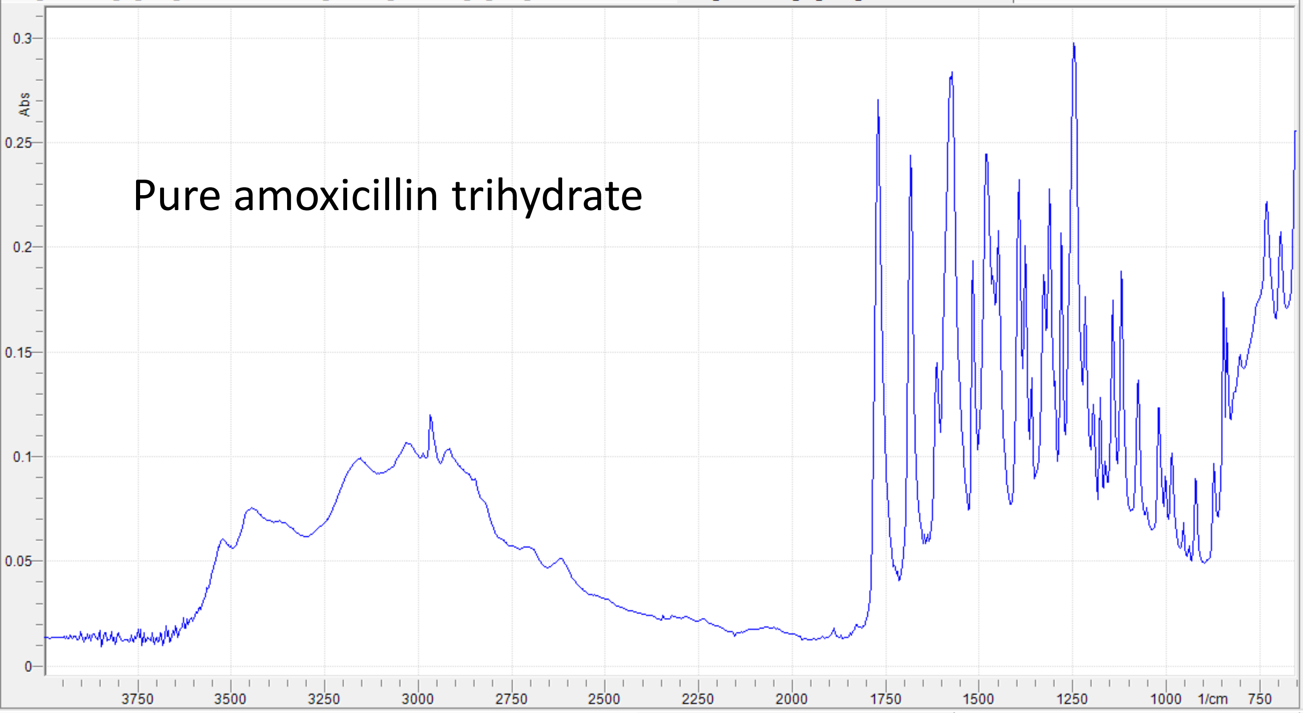
Pure amoxicillin trihydrate
Abs
0.3
0.25
0.2
0.15
0.1
0.05
0
3750
3500
3250
3000
2750
2500
2250
2000
1750
1500
1250
1000
1/cm
750

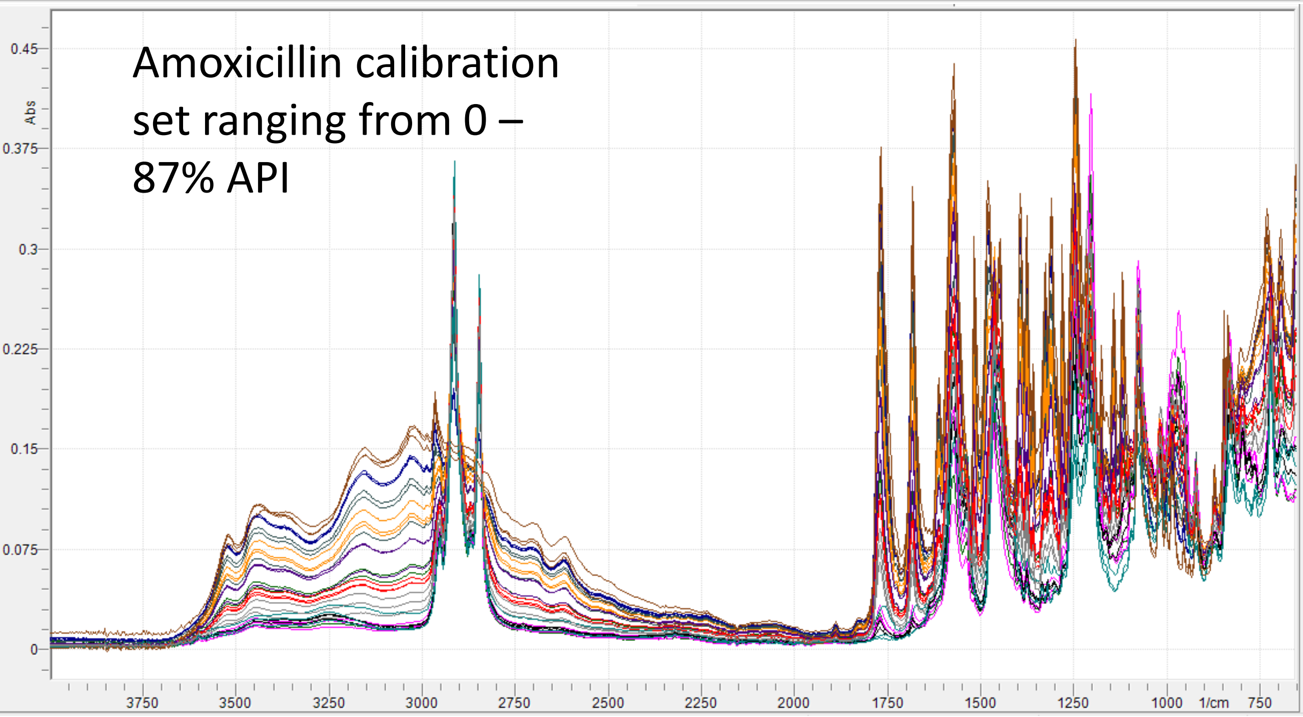
Amoxicillin calibration set ranging from 0 – 87% API
Abs
0.45
0.375
0.3
0.225
0.15
0.075
0
3750
3500
3250
3000
2750
2500
2250
2000
1750
1500
1250
1000
1/cm
750

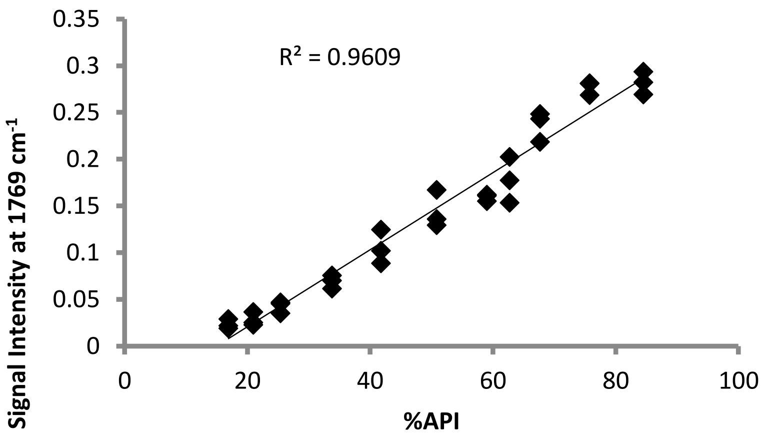
R² = 0.9609
Signal Intensity at 1769 cm⁻¹
%API
0.35
0.3
0.25
0.2
0.15
0.1
0.05
0
0
20
40
60
80
100
